# Effects of morphology and size of nanoscale drug carriers on cellular uptake and internalization process: a review

**DOI:** 10.1039/d2ra06888e

**Published:** 2022-12-20

**Authors:** Wenjie Zhang, Reza Taheri-Ledari, Fatemeh Ganjali, Seyedeh Shadi Mirmohammadi, Fateme Sadat Qazi, Mahdi Saeidirad, Amir KashtiAray, Simindokht Zarei-Shokat, Ye Tian, Ali Maleki

**Affiliations:** Department of Nuclear Medicine, West China Hospital, Sichuan University No. 37, Guoxue Alley Chengdu 610041 Sichuan Province P. R. China; Catalysts and Organic Synthesis Research Laboratory, Department of Chemistry, Iran University of Science and Technology Tehran 16846-13114 Iran Rezataheri13661206@gmail.com R_taheri94@alumni.iust.ac.ir maleki@iust.ac.ir +98 21 73021584 +98 21 77240640-50; Department of Orthodontics, West China Hospital of Stomatology, Sichuan University No. 14, 3rd Section of South Renmin Road Chengdu 610041 P. R. China

## Abstract

In the field of targeted drug delivery, the effects of size and morphology of drug nanocarriers are of great importance and need to be discussed in depth. To be concise, among all the various shapes of nanocarriers, rods and tubes with a narrow cross-section are the most preferred shapes for the penetration of a cell membrane. In this regard, several studies have focused on methods to produce nanorods and nanotubes with controlled optimized size and aspect ratio (AR). Additionally, a non-spherical orientation could affect the cellular uptake process while a tangent angle of less than 45° is better at penetrating the membrane, and *Ω* = 90° is beneficial. Moreover, these nanocarriers show different behaviors when confronting diverse cells whose fields should be investigated in future studies. In this survey, a comprehensive classification based on carrier shape is first submitted. Then, the most commonly used methods for control over the size and shape of the carriers are reviewed. Finally, influential factors on the cellular uptake and internalization processes and related analytical methods for evaluating this process are discussed.

## Abstract 2

In the field of targeted drug delivery, the effects of size and morphology of drug nanocarriers are of great importance and need to be discussed in depth.

## Graphical abstract

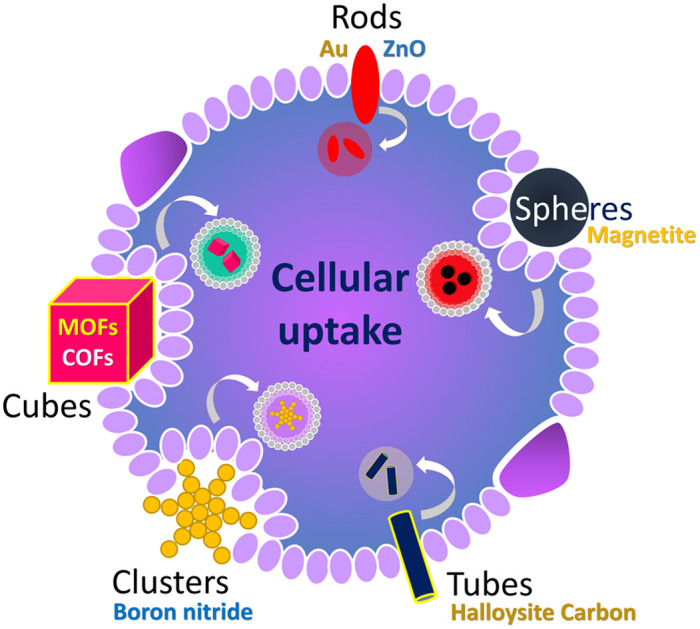

## 1. Overview

The rapid development and progress in nanotechnology and its related applications have opened great new windows to profoundly consider influential aspects in drug delivery and cancer cell treatment over past decades.^1^ Cancer is the second biggest mortality factor worldwide and cancer patients do not die only as a cause of the primary tumor, but their lives are threatened by the systemic impacts of metastases on other organs apart from the leading site.^2^ On the other hand, the morbidity and mortality of cardiovascular diseases (CVDs) are the highest among all illnesses worldwide, and they have emerged as a significant global public health issue.^3^ The creation of medications for the treatment of CVDs has been elevated to the top of the priority list in light of this dire scenario.

According to the parameters referred to above, the focus on targeted drug delivery systems has grown.^4^ There have been various studies on drug carriers utilized in drug delivery systems with diverse characteristics in recent decades.^5–11^ Among them all, nanocarriers are a broad subset that has attracted much more attention because of their small and nanoscale dimensions, which leads to better loading and uptake into the cell,^12^ and can be applied in many fields, such as catalysis,^13–39^ photocatalysis,^40–42^ solar cells,^43–45^ and environmental aspects.^46–49^ This advantage also enhances smart and targeted drug delivery.^50^ Nanoscaled small particles can improve drug concentration inside a tumor with privileged aggregation based on the large pore size and flawed lymphatic system of the neovasculature, described as upgraded permeation and retention efficiency.^51^ Additionally, nanotechnology-based drug delivery is a novel way to overcome the therapy bottleneck for cardiovascular disease due to the fast growth of nanoscience and the exceptional performance of nanomaterials. A class of nanomaterials known as nano-drug delivery systems (NDDSs) can increase the stability and water solubility of drugs, lengthen the cycle time, boost the rate at which drugs are absorbed by target cells or tissues, and decrease enzyme degradation, all of which contribute to increased drug safety and efficacy.^52,53^ NDDSs have greater absorption and can be administered *via* various methods, including oral or intravenous delivery. Nanomaterials will have a greater chance of interacting with blood vessels, blood, and their constituent parts, which will have a significant effect on human health.

Many nanomaterials have been used, such as nanoparticles (NPs), nanocrystals, nanocapsules, and nanotubes. Among these cases, NPs are the best-known nanomaterial, split into two significant subsets: organic NPs and inorganic NPs.^54^ Liposomes, polymeric micelles, and cellulose nanocrystals (CNCs) are some examples of organic NPs.^55^ At the same time, quantum dots (QDs), gold NPs (GNPs), silver NPs (SNPs), carbon nanotubes (CNTs), and mesoporous silica NPs (MSNs) are considered inorganic NPs.^56^ Nevertheless, modifying the morphology and size to keep organic aggregations under an assured size limit is challenging, especially in living systems.^57^ Unlike organic NPs, drug carriers based on inorganic NPs have enhanced surface modification and size tunability. Additionally, some inborn physicochemical features in inorganic NPs, such as converting irradiated energy to heat or toxic radicals leading to elevated temperature, and photothermal treatments in the case of solid tumors, are confirmed. Additionally, they are not sensitive to the body's environment.^58^ As a substantial downside restricting pharmacological utilization, the *in vivo* degradation of inorganic NPs is a problem, harming normal cells.^59^ However, due to the advantages of these NPs, their traces in pharmacological fields are always being felt. To advance the employment of these nanocarriers, surface modification agents should be substituted, including polyethylene glycol (PEG), leading to PEG-conjugated nanocarriers or surface-covered MSNs.^60^

As a temporary biomolecule found in nature, messenger RNA (mRNA) facilitates the translation of genetic information from genes encoded in DNA to proteins dispersed throughout the cell.^61^ Fortunately, several carriers, and biomaterials developed for DNA and small interfering RNA (siRNA) have shown signs of hopeful development as a basis for mRNA delivery methods. mRNA has been studied for more than 50 years. However, because of its apparent instability, susceptibility to degradation, poor translatability, and immunostimulatory effects, its extensive use in medical research and the creation of innovative treatment modalities have been restricted.^62^ Due to advances in knowledge of the mRNA structure and its connection to mRNA stability, as well as the creation of several chemical modification techniques, these problems have primarily been overcome.^63^

Following these discoveries, it has become easier to synthesize mRNA with various structural changes (such as anti-reverse cap analogues (ARCA), 3′-globin UTR, and a poly-A tail), which nevertheless have functional activity for gene-based and immunotherapy treatments. The potential for using mRNA as a therapeutic tool is now becoming a reality because of these developments in stability and usefulness.^64^ However, much like other nucleic acids (such as DNA and siRNA), naked mRNA is unable to easily pass the cell membrane on its own. It must be delivered using additional molecules to improve cell penetration.^65^ Although utilized as mRNA carriers, viral vectors may suffer from their possible immunological side effects, toxicity, and vector-size restrictions.^66^ More research has been done on non-viral techniques, such as electroporation, gene guns, and sonoporation as mRNA delivery methods.^67,68^ Although technically possible, utilizing such methods to manipulate cells *ex vivo* using mRNA transfection is time-consuming, costly, and generally unsuitable for widespread application.^68^ Biomaterials have shown remarkable promise for transporting different biomacromolecules, including DNA and siRNA, with significant improvement over the non-viral methods listed above. Biomaterials are more biocompatible and diverse than *ex vivo* technologies or viral vectors. They can be easily synthesized for efficient *in vivo* administration and the regulated release of medicines.^69^ For instance, protamine has shown considerable improvement in mRNA's capacity for transfection, and various protamine–mRNA complexes are currently being tested in clinical studies on cancer patients.^70^

Various nanocarriers have been synthesized in a diverse and broad spectrum of shapes, *viz.* spherical, rod, tube, star, cube, cluster, disc, flower, needle, star, spindle, and even unusual morphologies like square, wrinkled, octahedral, *etc.*^71^ For instance, Kang *et al.* have produced an amphiphilic copolymer based on cyclic parts with a dumbbell morphology, demonstrating upgraded cell uptake.^72^ Additionally, different methods for synthesizing NPs, from conventional hydrothermal and sol–gel to complex synthesis routes, have been introduced.^73^

Controlling the size and shape of NPs leads to smart NP selection for cell penetration and internalization, which has a further impact on NP accumulation on cancerous cell spots and smart and stimuli-responsive drug release.^74^ Abundant design regulations have been suggested to control the characteristics of NPs by the participation of various reactants, stabilizing and capping agents or ligands, with diverse reaction conditions, such as temperature, pH, solvents, and concentration.^75^ Choi *et al.* claimed that, for the size-controlling production procedure of crystalline silicon NPs, a novel self-assembly approach requiring a controlled low plasma ion energy close to sputtering threshold energy in the rare gas reaction environment is needed. The resulting NPs showed an accelerated rate of nucleation and growth, and their nanoscale size is appropriate for plasma ion energy. Conversely, previous studies entailed preparing high-temperature conditions, hydrogen fluoride (HF) solution, and further procedures to ensure NP uniformity on their support.^76^ On the other hand, as reported by Huynh, chitosan (CS) has a leading role as a shape-directing agent due to its green and nontoxic nature in contrast to toxic capping agents like cetyltrimethylammonium bromide (CTAB) and hexadecyltrimethylammonium chloride (CTAC).^77^

The cellular uptake of drug-loaded nanocarriers commonly follows an endocytosis mechanism influenced by the physicochemical features of NPs. In a recent study, it was reported that antibody antigen-binding sections could enhance the uptake of NPs in the case of NPs smaller than 10 nm.^78^ TEM, SEM, and Raman microscopy methods evaluate and discriminate the cellular uptake and internalization of NPs. In addition, transmission electron microscopy (TEM) and scanning electron microscopy (SEM) are considered destructive imaging methods, while the least sample preparation is needed in Raman microscopy. Besides, the Raman microscopy method provides both *in vivo* and *in vitro* cellular imaging to pursue cellular uptake and internalization.^79^

Herein, we intend to provide a brief report on the different types of nanocarriers applied to drug delivery systems with a comparative look at the various characteristics of nanocarriers, including their morphology and size. In this regard, a classification is presented based on the most commonly used materials and methods for synthesizing nanocarriers. Additionally, various approaches for controlling the size and shape of nanocarriers are assessed. The most recent research, new techniques, and advances are highlighted here. As an essential part of the review, the effects of nanocarriers on drug delivery for cellular uptake and internalization were investigated. Finally, various routes and devices have been applied to confirm and detect the drug loading, cellular internalization and tumor penetration, blood circulation, and even the drug release model. Nanocarriers are the optimal vehicles to contribute to drug delivery applications.

## 2. Classification of drug carriers based on morphology

Drug delivery carriers are the most significant part of drug delivery systems in medical treatment and smart drug delivery with specialized targets in the human body. Here, a general classification of drug carriers is proposed with a comprehensive overview of NP morphology.

### 2.1. Spherical-shaped nanocarriers

Since most therapeutic nanocarriers are spherical, categories of nanostructures can be roughly separated into spherical and non-spherical. Spherical nanocarriers come in various shapes, from micellar to vesicular, which include internal structures with a hollow center and two continuous layers.^80^ Barua *et al.* reported higher non-specific cellular uptake of polystyrene (PS)-based nano-spheres by breast cancer cells (BT-474, SK-BR-3, and MDA-MB-231) compared with their nanorod and disc counterparts.^81^ However, after covering them with the monoclonal antibody trastuzumab, it was shown that nanorods were more capable of being taken up by cells than spheres or discs. This was attributed to the increased trastuzumab adsorption caused by the nanorods' larger surface area per unit volume. Therefore, the applicability of rod-shaped carriers is preferred to other morphologies, considering their enhanced cellular uptake and increased antibody adsorption. Additionally, scientists have shown that rod and spherical structural stiffness plays a part in cellular internalization. An inverse relationship between stiffness and cellular internalization for rod-shaped PS-based devices has been authenticated. However, spherical particles did not have any effect on cellular internalization with a change in stiffness.^82^ On the other hand, it has been noted that cellular absorption of nanostructures is cell-type-specific. According to Agarwal *et al.*, mammalian epithelial cells and immune cells choose disc-shaped nanostructures with high ARs (2–3) over those with lower ARs (∼1).^83,84^ Therefore, AR increases as NPs move from spherical (aspect ratio = 1) to elongated structures (aspect ratio > 1). However, some scientists have defined the AR of a discoidal particle as the ratio of the particle's diameter to its height (the ratio of the secondary axis length to that of the major axis), demonstrating a reciprocal relation with the AR of a discoidal particle. Generally, particles that have similar secondary but various major axes were prepared to explore the AR effect. It is indicated that AR might affect the hemodynamic forces applied to particles along with systemic circulation, the particles' intratumoral diffusion coefficient, and the particles' uptake rate by tumor cells.

*In vivo*, the cellular absorption of nanostructures in different organs is shape- and cell-type-dependent. In the aforementioned study by Yi *et al.*, it was shown that mice preferentially absorbed nanostructures of various morphologies after intravenous injection. Compared to spherical nanostructures like polymersomes and micelles, cylindrical micelles (filomicelles) in this study showed increased uptake by monocytes, granulocytes, neutrophils, and macrophages in the blood. However, when it came to forming associations with immune cells in the spleen and liver, spherical nanostructures outperformed filomicelles.^85^ Despite the evidence that the geometrical aspects of nanostructures influence cellular uptake, one should exercise caution about making too many generalizations. However, the preferred morphology would differ based on the cell type.

### 2.2. Rod-shaped nanocarriers

**Fig. 1:**
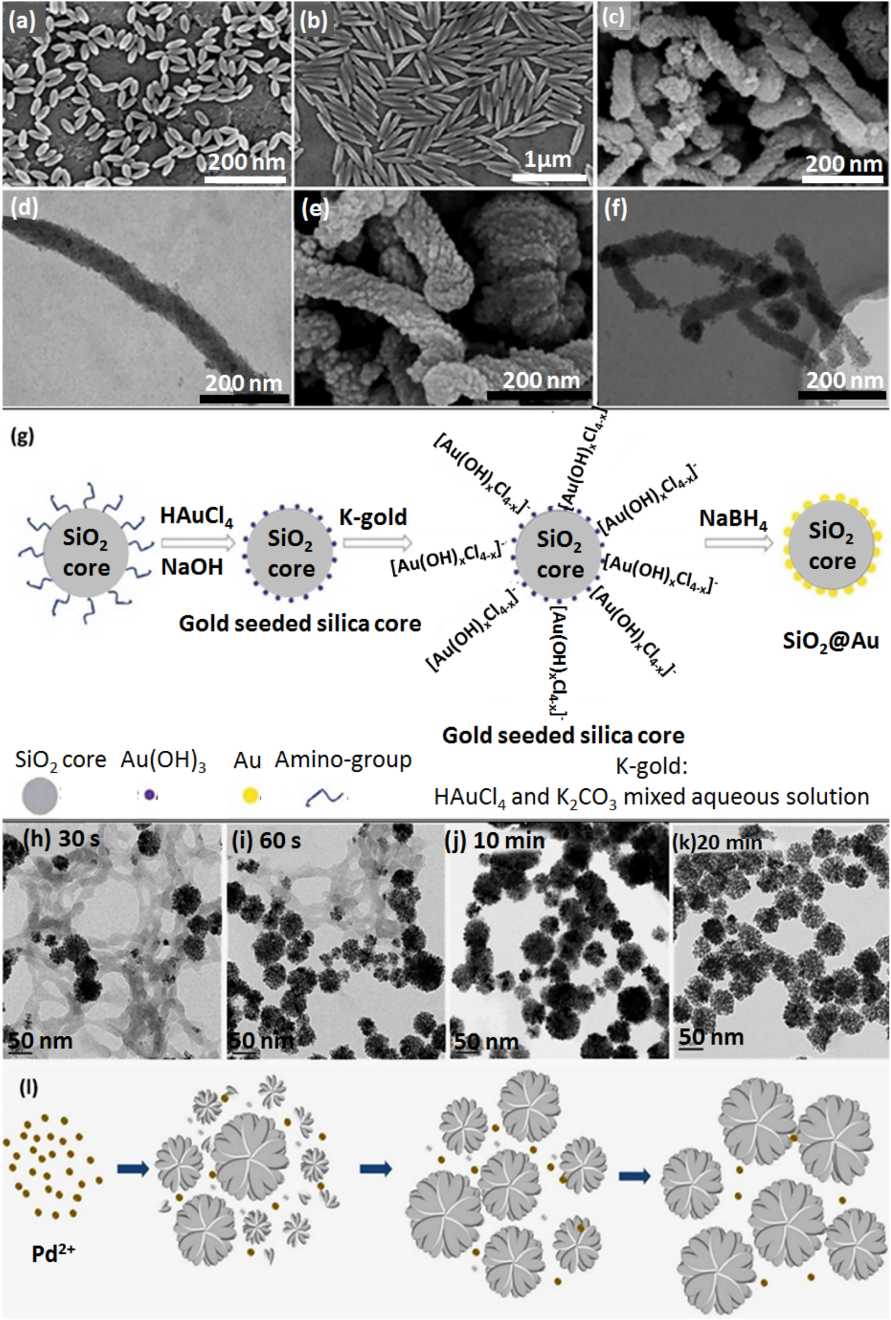
The SEM of NPs: (a) rod-shaped PS with AR:2 (2AR-PS-R), (b) rod-shaped PS with AR:5 (5AR-PS-R). Figures (a) and (b) were adapted by permission from: *ACS Biomater. Sci. Eng.*, 2020, **6**, 4916–4928.^89^ (c) SEM image of rod-shaped MSNs, (d) TEM image of rod-shaped MSNs, (e) SEM image of rod-shaped amino-functionalized MSNs, (f) TEM image of rod-shaped amino-functionalized MSNs. Figures (c)–(f) were adapted by permission from: *Microporous Mesoporous Mater.*, 2020, **294**, 109896.^90^ (g) The synthesis procedure of the SiO_2_@Au NPs. Figure (g) was adapted by permission from: *RSC Adv.*, 2020, **10**, 33119–33128.^114^ (h)–(l) Synthesis process of TEM images of CS-stabilized porous flower-shaped Pd NPs with quenched reduction after (h) 30 s, (i) 60 s, (j) 10 min, (k) 20 min of reaction time. (l) A plausible formation procedure of CS-stabilized porous flower-shaped Pd NPs. Figure (h)–(l) were adapted by permission from: *Carbohydr. Polym.* 2019, **205**, 340–352.^115^

Among this spectrum of broad and differing morphologies, rod-shaped nanocarriers have attracted researchers' attention due to their narrower cross-sectional area, which leads to rapid loading into the cell.^86^ Rod-shaped GNPs have generally been synthesized *via* a seed-assisted growth approach. Based on the anisotropy of specific rod-shaped GNPs, two individual transversal and longitudinal surface plasmon resonance (SPR) bands come into view in the visible and near infra-red (NIR) regions, respectively. Due to the photothermal effect, the SPR bands turn into heat, leading to high-temperature and irretrievable cancer cell lesions.^87^ Besides, compared with nanosphere GNPs, nanorods display a considerable improvement in fluorescence. This enhancement is because of the adjustability of the localized surface plasmon resonance (LSPR) of nanorod GNPs to fit the spectrum of the red/NIR dye. This efficiently enhances the fluorescence of red and NIR dyes to obtain optimum improvement in fluorescence. Nanorod GNPs with upgraded red/NIR emission are superb bioimaging candidates.^88^ In a recent study, Mitragotri *et al.* prepared different nanocarriers to elucidate the role of carrier parameters on particle uptake and transmission through the blood–brain barrier (BBB). The results indicated that rod-shaped PS NPs, as seen in Fig. 1a and b, show slower and less efficient endocytosis by increasing the AR.^89^ Additionally, the lower elastic moduli of the particles make them potentially more readily deformable than stiffer NPs. This deforming ability of ‘soft’ nanogel and hydrogel particles is thought to elucidate the extended circulation half-life and diminished accumulation in gross filtration organs, such as the spleen, but enhanced accumulation in organs with tiny capillaries like the lungs and brain.

In a recent study by Li *et al.*, amino-modified MSNs (amino-MSNs) with a wrapped rod-shaped morphology were synthesized through a biomimetic approach to carry an ibuprofen (IBU) drug with poor water solubility. The amino-MSNs were employed as oral delivery drug nanocarriers. A comparative study was applied to investigate *in vivo* and *in vitro* applicability of functionalized MSNs compared to pristine MSNs. Amino-MSNs exhibited a high drug loading amount (29.12%) according to the firm hydrogen bonding force between the amino functionalization groups and IBU's carboxylic acid part. As observed in Fig. 1, panels c and e, synthesized structures were monodispersed with a defectless wrapped rod morphology. MSNs had circa 100 nm outer diameter and 700 nm length. TEM images of the pristine MSNs with two margin types are depicted in Fig. 1, panel d. Notably, the amino-MSNs with 70 nm outer diameter, 500 nm length, and one margin type are demonstrated in Fig. 1, panel f.^90^

Interestingly, according to morphology and size influence on blood half-life, rod-shaped micelles exhibit much longer blood circulation and boosted tumor penetration compared to spherical ones.^91,92^ Among abundant rod-shaped GNPs, Zhang *et al.* chose a biosynthesis route utilizing the Pb^2+^-induced fungus *Aspergillus* sp. Morphology exchange during the preparation process took place from spherical GNPs to completely uniform rod morphology, displaying good attachment on mycelial surfaces.^93^

MSNs, carbon dot composites, nanohydroxyapatite, polymers, metal oxide NPs, and cellulose nanocrystal systems are some examples of carriers synthesized with rod morphologies.^94^ Another study reports that stabilized drug loading and particle sizes with a larger surface area to volume ratio show the most rapid release.^95^ Additionally, the tendency to use rod-shaped periodic mesoporous organosilica in multi-compartment structures to improve drug loading content and silica NPs with virus morphology to upgrade nano-bio conjugations has grown.^96^

The biocompatible, stable, flexible composition of synthetic hydroxyapatite combined with MNGs, has made these structures favorable for drug delivery applications. This work applied a hydrothermal method to produce magnetic nanoparticle (MNP)-decorated rod-shaped hydroxyapatite with cetyltrimethylammonium bromide (CTAB) surfactant as a shape-directing agent.

The synthesized iron oxide-hydroxyapatite was further loaded with curcumin anticancer drug (Cur@IO-HA) to investigate the efficiency of cellular uptake. Due to the abovementioned considerations, the prepared nanocomposite revealed hemocompatibility and excellent cellular uptake compared with free curcumin.^97^ Another study directed by Ramezani *et al.* showed the co-delivery of a gene (survivin shRNA) and a drug (camptothecin) simultaneously by PEGylated rod-shaped MSNs with diameter 100–150 nm. Camptothecin was first encapsulated by rod-shaped MSNs. Next, the PEGylation procedure of camptothecin-loaded NPs took place. Then, the condensation of iSur-DNA with a C/P ratio of 6 resulted in the formation of PEG@MSNR-CPT/Sur. After that, the AS1411 DNA aptamer was attached to as-prepared NPs (Apt-PEG@MSNR-CPT/Sur) to provide higher specificity for colorectal adenocarcinoma treatment. Finally, the Apt-PEG@MSNR-CPT/Sur system was efficiently taken up by the cell and demonstrated the controlled release of camptothecin and notable tumor repression in C26 tumor-bearing mice compared to PEG@MSNR-CPT and PEG@MSNR-CPT/Sur.^98^ Importantly, the prepared aptamer-attached drug carrier with a rod morphology provides target specificity *via* an active targeting procedure while having a high AR, which aids in the easy penetration of the rod-shaped nanocarrier into the target cell.

Scientists have examined the effect of the shape of nanoparticles on their *in vivo* pharmacokinetics to improve targeting efficiency, revealing that nanoparticle shape plays a key role in the internalization, retention, and penetration processes.^99^ However, studies on the effect of shape remain scarce and incomplete. Nanoparticles with different shapes show different characteristics in circulation, biodistribution, and cellular internalization processes. For example, worm-like nanoparticles can achieve wide internalization and long circulation.^100^ Nanorods with high AR achieve greater cell internalization than their spherical counterparts, but still less than that of nanorods with medium AR.^101^ Filaments (8 μm in length) are retained in the blood circulation ten times longer than their spherical counterparts. This phenomenon occurs because rapid flow induces strong hydrodynamic shear, making it difficult for long worm-like micelles to prolong their interaction with the cell surface.^102^ Hence, in the case of strong hydrodynamic shear, spherical carriers have a better chance in the competition between worm-like filaments and their spherical counterparts.

### 2.3. Star-shaped nanocarriers

Generally, two conventional synthesis routes (arm-first and core-first) for star polymers have been introduced. The core-first method is based on growing the polymerization process on a hetero-multifunctional core. In the arm-first approach, all individual polymeric arms are synthesized separately before connecting them to the core molecule.^103^ Higher scales of spherical and rod-like GNPs with increased cellular uptake have been reported compared to star-shaped ones. Compared to their linear counterparts, star polymers show enhanced drug loading content (DLC), drug loading efficiency (DLE), and longer circulation time.^104–106^ As computational studies reveal, a simulated drug delivery system for water-soluble drugs with droplets containing nanoparticle liquid crystals in different shapes has demonstrated that drugs are concentrated in the core of star-shaped NPs (red color). In contrast, the five star arms (blue color) do not carry drugs.^107^

### 2.4. Tube-shaped nanocarriers

Tube-shaped nanocarriers, specifically CNTs, can be chemically functionalized *via* covalent or non-covalent interactions, including π–π stacking. Because of the high specific surface area of single-walled CNTs (SWCNTs), they have higher drug-loading capability than liposomes or dendrimer drug carriers.^108^ Pristine SWNTs tend to accumulate in reticuloendothelial system organs for several days, resulting in damage to the reticuloendothelial system. Conversely, the removal of functionalized SWNTs occurs a few hours after administration with very low accumulation and toxicity in the reticuloendothelial system.

Furthermore, Kushwaha *et al.* utilized halloysite nanotubes (HNTs) with outer silica, and inner alumina layers and 50 nm diameter with biocompatibility, large AR, and enhanced AR mechanical strength that match drug delivery requirements.^109^ Generally, tubular surface structures are prepared based on the quaternary CTAB cationic surfactant.^110^

### 2.5. Cluster-shaped nanocarriers

Nanoclusters are other vehicles to carry drugs into target cells. Boron nitride (BN) nanoclusters are one example with remarkable chemical stability.^111^ Although many pieces of research have been devoted to cluster-shaped nanocarriers, magnetic nanoclusters (MNCs) of Fe_3_O_4_ and gold nanoclusters afford suitability for drug delivery applications.^112^ Another study has reported a controllable assembly of an amphiphilic copolymer, entitled poly(styrene-*co*-maleic anhydride) (PSMA), to form upconversion NPs (UCNPs) to heal large cancer tumors and discusses cell permeation. Oleic acid (OA) ligands were used to encapsulate UCNPs.^113^ The frequency dependence of LSPR on the size, shape, and dielectric surroundings of GNPs indicates that as the alignment of GNP clusters becomes closer, the particle's coupling matters, especially in surface-enhanced Raman scattering (SERS) studies and the nanogap notably upgrades the near-field electromagnetic field. As depicted in Fig. 1g, in a k-gold (K_2_CO_3_–HAuCl_4_) solution, the hydrolysis of HAuCl_4_ forms [Au(OH)_*x*_Cl_4−*x*_]^−^ where *x* depends on the hydrolysis amount.^114^

### 2.6. Flower-shaped nanocarriers

Nanocarriers with various flower morphologies are another attractive nanocarrier shape group for drug delivery and uptake.^116^ An organic–inorganic hybrid nanoflower (hNF) system was prepared based on glucose oxidase and copper ions immobilized in amine-functionalized magnetic NPs (MNP-GOx NFs). The antibacterial property was obtained by bacterial cell distribution with H_2_O_2_ generated from GOx.^117,118^

The CS stabilizer acts as a multifunctional material for Pd NP synthesis.^115^ The green synthesis of flower-shaped porous Pd NPs was conducted by utilizing different ratios of CS and vitamin C (reducing agent for Pd^III^ to Pd^0^). After production of Pd nuclei under the impact of vitamin C, the presence of CS played a crucial role in producing Pd NPs with a flower morphology by covering the NPs through intense interactions with Pd nuclei. The CS layer leads to anisotropic directed growth for Pd nuclei, resulting in the porous flower shape. An increase in CS concentration causes smaller Pd NPs. According to this principle, the favorable size of Pd NPs could be controlled by monitoring the concentration and the layer formation rate of the CS (Fig. 1h–l).^115^ Spherical Pd NPs with a plasmonic effect are more sensitive to variations in refractive index than other morphologies.^119^

### 2.7. Spindle-shaped nanocarriers

**Fig. 2:**
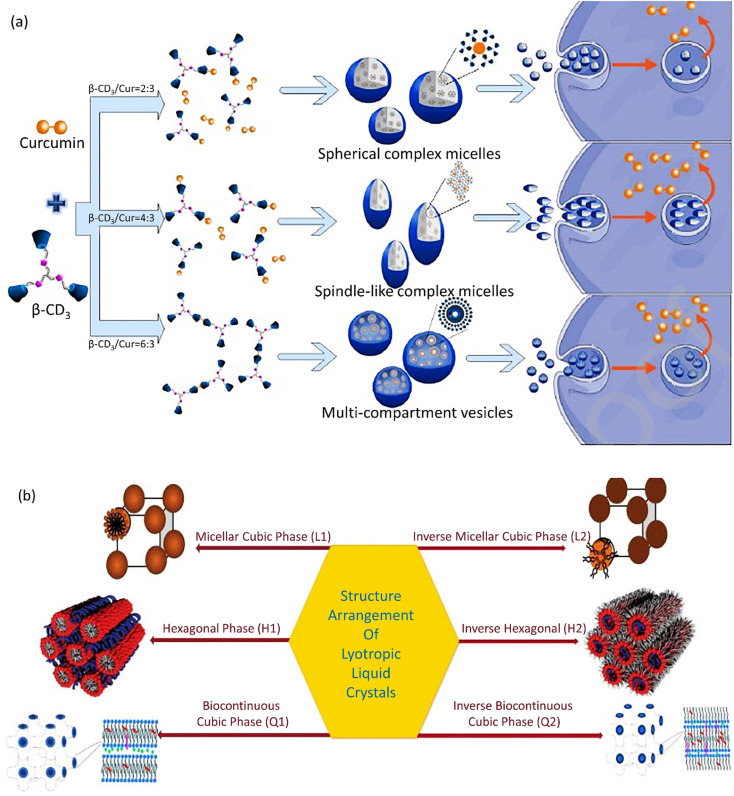
(a) Supramolecular self-assemblies with various shapes based on curcumin and β-CD_3_. Figure (a) was adapted by permission from: *Carbohydr. Polym.*, 2020, **231**, 115714.^120^ (b) Different phases of lyotropic liquid crystals. Figure (b) was adapted by permission from: *J. Pharm. Res. Int.*, 2021, 118–135.^122^

Supramolecular self-assemblies based on β-cyclodextrin (β-CD) are a subunit of nanocarriers utilized in cancer treatment. A supramolecular self-assembly system with a β-cyclodextrin trimer (β-CD_3_) as a host and a curcumin anticancer drug as the guest was prepared. With an increase in β-CD_3_ ratio, diverse supramolecular self-assembled carrier morphologies were obtained from complex spherical micelles to spindle-shaped complex micelles and then to multi-compartment vesicles (Fig. 2a). Consequently, complex micelles with a spindle morphology demonstrated improved cellular uptake and apoptosis. The spherical complex micelles emerged with the least efficient performance.^120^ The high cell uptake and boosted internalization of CNCs is related to an accumulation of spindle-like CNCs with an increased AR in cancerous cells in comparison with spherical NPs.^121^

### 2.8. Needle-shaped nanocarriers

Leukocyte membrane-coated gallium nanoswimmers††Converts different kinds of energy into mechanical motion. are needle shapes that can be prepared *via* the pressure-filter-template process.^123^ Another piece of research stated that hydroxyapatite NPs with needle morphology could diffuse into the cell membrane more conveniently than other morphologies. The mean size of the spherical NPs was evaluated as 54 nm, and the length and width of needle-shaped NPs were 246 nm and 43 nm, respectively. Hydroxyapatite NPs with needle morphology at an optimum concentration of 100 mg L^−1^ can better diminish cell growth and reproduction by more than 73%.^124^ Moreover, polyamidoamine (PAMAM)-CNC nanocarriers conveniently penetrate the cell and are internalized due to their particular needle morphology.^125^

### 2.9. Cubic-shaped nanocarriers

Cubosomes‡‡Lyotropic liquid crystals as soft NPs with isotropic lipidic features reinstated with stabilizing agents, *viz.* poloxamers (F127, F108). show inimitable characteristics, including a specific cubic shape, which facilitates their incorporation with lipophilic, hydrophilic, and amphiphilic pharmaceutics.^126^ The cooperative incorporation of amphiphilic lipids in cubic-shaped nanocarrier formation has attracted a lot of consideration for the following reasons: first, in special physiological conditions, these lipids self-assemble into well-formed biomimetic NPs, including cubosomes with the abovementioned superiorities. Second, these nanocarriers highly protect the drugs and active species from degradation and, thus, lead to an efficient controlled release. Third, the gradual amphiphile concentration and increase in temperature permit diverse structural arrangements, *viz.* micellar cubic phase (I_1_), hexagonal phase (H_1_), lamellar phase (Lα), and bicontinuous cubic phase (Q_1_), as illustrated in Fig. 2b.^127,128^

### 2.10. Disc-shaped nanocarriers

Preparation approaches for disc-shaped nanocarriers mainly include deformation procedures from spherical morphologies by time-consuming methods alongside external stimuli. The particle's diameter and thickness ratio determine the disc's morphology.^129^ Nanodisc and nanorod hydrogels were prepared to investigate their cell uptake. The obtained results show that disc-shaped hydrogels had higher cellular uptake than nanorods.^130^ Disc-shaped MSNs with a large surface area due to porosity emerged with higher cell uptake than spherical counterparts.^131,132^ Concise information about the preparation conditions, the material's origination, and shape and size of the nanocarriers are presented in Table 1.

**Table 1:** Brief information on various morphologies of nanocarriers

Entry	Nanocarrier system	Technique	Morphology	Size (nm)	Ref.
1	Lignin and CNCs^a^	Acid hydrolysis (60 vol% + 25 vol% HCl)	Needle	276 ± 45.7 (length), 17.5 ± 4.52 (width)	133
2	CNCs	Acid hydrolysis (64–65 wt% H_2_SO_4_)	Needle	323 (length), 7 (width)	134
3	Fe-modifies AuNPs	Brust–Schiffrin	Spherical	5.6	135
4	MSNs^b^	Sol–gel	Spherical	49.73	136
5	MSNs	Sol–gel	Spherical	3–40	137
6	Ni-based NPs supported on fibrous silica nano-spheres	Hydrothermal	Spherical	26.3–48.8, 24.0–82.3	138
7	MSNs	Green chemistry	Spherical	25.6–54.6	139
8	MSNs	Green chemistry	Spherical	200–250	140
9	MSNs	Green chemistry	Spherical	32–85	141
10	Carbon dots/hydroxyapatite nanocomposite	Hydrothermal	Spherical	142	142
11	Amphiphilic cyclodextrin	Self-assembly	Spherical	100	143
18	Zinc ferrate NPs	Hydrothermal	Spherical	150	144
			Litchi-like	120	
			Raspberry	50	
20	CNCs	Acid hydrolysis (62 wt% H_2_SO_4_), enzymatic hydrolysis using Cellic CTec 2 (Novozymes), enzymatic hydrolysis using Cellic CTec 2 (Novozymes)	Needle	<10 (width), 6–12 (width), 14–22 (width)	145

a Cellulose nanocrystals.

b Mesoporous silica NPs.

The fact that the tiniest blood veins have a 4 μm diameter should be considered when determining particle size, since a micrometer-sized object might result in an embolism. Therefore, an acknowledgement of size should consider both the physical impacts of the size once it is within the body and its capacity for internalization.^146^ The likelihood of internalizing particles smaller than 100 m in human cells, which range in size from 1 to 100 μm, is consequently negligible. Cells with sufficient space to accommodate the particle are within this particle size range for intracellular delivery. Experimental evidence supsports better bioavailability of the endocytosed drug carrier at 100–1000 nm^147^ in addition to the higher rates of endocytosis of smaller NPs (<100 nm).^148^ According to several studies, within the range of 1–100 nm, 50 nm NPs have the highest level of cellular uptake, with 14–20 nm NPs having a greater endocytotic rate than 100 nm NPs.^149^

The NPs did not demonstrate a significant difference in cellular uptake between 25 and 130 nm, despite some findings claiming that NP internalization is stronger between 50 and 100 nm.^150^ On the other hand, it was found that 95–200 nm is the optimal size for enhanced cellular absorption in a study on thio-organosilica NPs of 50–500 nm.

Poly(lactic-*co*-glycolic acid) (PLGA) microparticles with 6.5 ± 3.9 μm size still adhered to the cell surface after 4 hours and needed extra time for endocytosis.^151^ Within the same timeframe, PLGA NPs of size 389 nm (polydispersity index = 0.2) had already been endocytosed into the intracellular compartment and encased in vesicles. These findings were in line with research done by Loh *et al.*, which showed that NPs with diameters between 110 and 390 nm were considerably more readily absorbed than chitosan particles that were >1 μm in size.^152^ Intriguingly, PEG particles <5 μm can enter cells *via* pinocytosis despite the fact that they do not do so as quickly as NPs.^153,154^ Similar to the size of an aggregated nanocluster being greater than that of a single NP, taking the probability of NP aggregation into account will have an impact on the pace of internalization.

Acosta's assessment^155^ of the literature mostly concluded that NPs less than <500 nm induce stronger cellular uptake than NPs that are bigger. These results were also in agreement with the improved penetration capabilities of the specific nanocarrier systems that held the NPs. Although the uptake of NPs smaller than 500 nm has reduced viability, it might be aided by employing a beneficial delivery method. A useful delivery method would ideally include lipophilic characteristics or an appropriate surface charge to improve internalization. Even though a particular piece of research advocates the use of smaller NPs (<100 nm), there are discrepancies in other studies that show how much particle size influences internalization and its complementary system. The variance in internalization patterns and NP size shows that the kind of cell also influences the impact of NP size and chemical makeup of the nanomaterial.^152,153^

The form of the NP was found to have a crucial impact in many of the experiments that followed in enhancing internalization. Chithrani *et al.*^156^ looked into the uptake of sphere- and rod-shaped gold nanoparticles. Their assertion that spherical particles have a greater likelihood of internalization is supported by the fact that the uptake of 74 nm and 14 nm spherical NPs was 500% and 375% higher than that of 74 nm and 14 nm rod-shaped NPs, respectively. It has been hypothesized that the variance determines cell surface binding in the curvature between the two forms. Unlike spherical NPs, rod-shaped NPs have a wider area of contact with the cell membrane receptors when they are in contact with the cell surface. As a result, they obstruct the remaining accessible membrane receptors, lowering the amounts of NPs internalized. Similar findings, which demonstrate that spherical particles internalize significantly more quickly than asymmetrically shaped particles, were published by Han *et al.*^157^

Considering the relationship between contact angle and particle internalization, rod-shaped NPs are more likely to do so when their primary axis is parallel to the cell membrane. The long axis of the rod aligning perpendicular to the cell will increase the internalization rate, and the rate will decrease as *ø* increases.^158^ This theory, based on how the NP is oriented with respect to the cell membrane, may also guide the creation of NP forms with several short features to facilitate internalization. Yang *et al.* reported another possibility through research involving different-shaped PEG-based PRINT (particle replication in non-wetting templates) particles. Nanocylinders were internalized significantly more than microcylinders and nanocubes among the various forms examined. It was hypothesized that the increased surface area, which enabled more multivalent ionic contacts with the cell membrane and allowed for endocytosis and phagocytosis, was responsible for the higher cell absorption.^159^

The strongest antibacterial activity of silver nanoplate NPs, compared to nanospheres and nanorods, was based on the larger surface area that binds with the bacterial cells. This theory demonstrated that mesoporous silica long-rod NPs had higher internalization and retention than spheres and short rods.^160^ Additionally, it has been demonstrated that distinct nanoshaped particles can accumulate in varied ways in different organ systems. Based on an analysis of the biodistribution data, the shape effect of silicon NPs, and their accumulation in particular tissues it was found that discoidal-shaped NPs tended to internalize more in the lung than spherical, cylindrical, or quasi-hemispherical NPs.^161^ The internalization of discoidal and quasi-hemispherical NPs was greatest in spleen tissue. In contrast, cylindrical NPs accumulated more in the liver than in the other three forms: the heart and the heart's chambers. Furthermore, nanoworms had higher tumor uptake in fibrosarcoma and breast cancer cell lines than spherical NPs. That irregular spherical NPs accumulated preferentially in the spleen, while regular spherical NPs accumulated in the liver further supported these findings.^162^

Contrary to the abovementioned facts, which suggest that non-spherical NPs have higher internalization, we cannot infer from the dynamic properties and unrivaled high surface area to volume ratio of spherical NPs that they are less prone to internalization. In fact, due to their higher ability to load drugs, spheres should be preferred over non-spherical NPs when assessing the therapeutic potential of NPs. We cannot dispute that all of the NPs under investigation do not have constant extra NP characteristics and that these other NP parameters would influence the pace of cellular absorption. Many scholars assert speculative hypotheses on internalization kinetics based on NP shape.

Recently, ionizable cationic lipids have played a fundamental role in creating modern gene therapies for different biomedical applications, including COVID-19 vaccines. Be that as it may, it remains vague whether the definition of lipid nanoparticles (LNPs) utilizing DLin-MC3-DMA, an optimized ionizable lipid clinically utilized for small interferometer RNA (siRNA) treatment, also encourages high liver-selective transfection of other gene treatments, such as plasmid DNA (pDNA). It was found that the DLin-MC3-DMA, DLin-KC2-DMA, or DODAP lipids already created for siRNA delivery took up an unexpected characteristic rank arranged according to gene expression efficiency when utilized for pDNA. Specifically, DLin-KC2-DMA simplified higher *in vivo* pDNA transfection compared to DLin-MC3-DMA and DODAP, conceivably due to its head group pKa and lipid tail structure. Interestingly, LNPs defined with either DLin-KC2-DMA or DLin-MC3-DMA showed higher *in vivo* protein production within the spleen than within the liver.^163^ Designing LNPs for lymph node targeting seems a challenging issue since the surface of LNPs ought to have a negative charge and a huge number of PEG chains to reduce the interactions with negatively charged lymphatic vessels and interstitium. However, on the other hand, it ought to have a positive charge and a low amount of PEGylation to advance interactions with immune cells at the lymph nodes.^164^

As drug carriers, especially at the nanoscale, have an unprecedentedly wide scope in drug delivery systems, the various morphologies and related characteristics of NPs should be highlighted. Accordingly, many smart drug delivery systems showed an upgraded performance assigned to a preference for a particular nanocarrier morphology. Meanwhile, the nanocarrier morphology selection would be optimized by considering cellular uptake and internalization efficiency. As deduced from this classification, based on the nanocarrier shape and entrance angle to the cell membrane, nanorod carriers are expected to perform better in scientific studies.

## 3. Control of nanocarrier size and morphology

There are diverse significant parameters of nanocarriers affecting their cellular uptake and internalization, such as size, shape, surface charge, surface functionalization, and the interactions between these factors. This section highlights the importance of controlling nanocarrier size and shape.

### 3.1. Size controlling

**Fig. 3:**
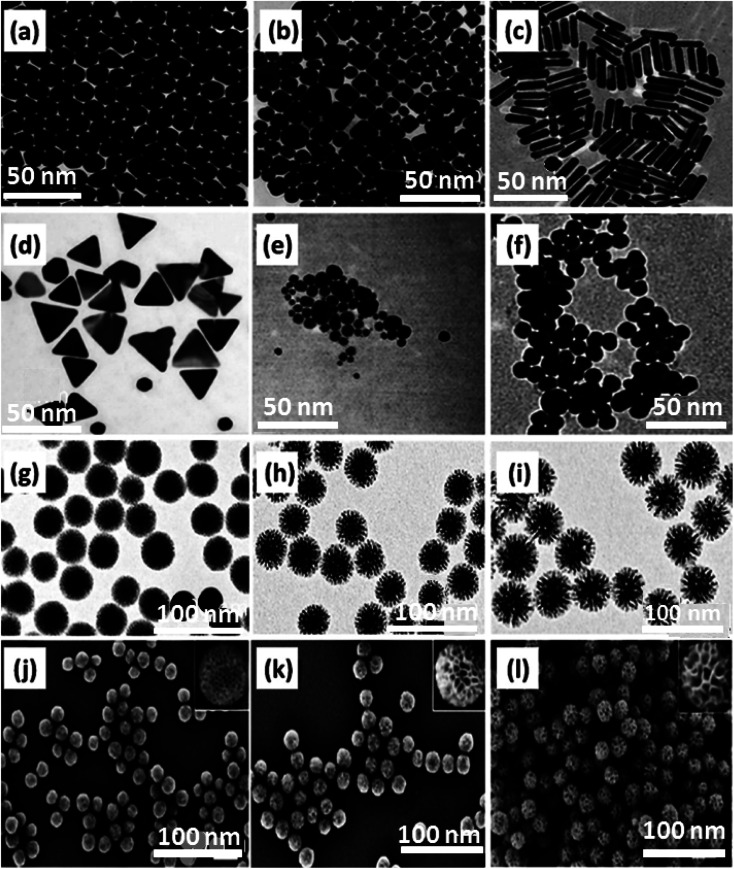
TEM images related to: (a) spherical GNPs [CTAB], (b) cubic GNPs [CTAB], (c) rod-shaped GNPs [CTAB], (d) prismatic GNPs [CTAB], (e) CTAB/citrate-spherical GNPs, (f) citrate-spherical GNPs. The scale bar of all the panels is 50 nm. Figures (a)–(f) were adapted by permission from: *ACS Omega*, 2019, **4**, 242–256.^165^ The morphology of DLMSN_FCA_ with various mass ratios of FCA to CTAB. (g)–(i) TEM images of DLMSN_FCA_ with different mass ratios of (g) 0.1, (h) 0.2, (i) 0.3. The inset images depict the direct relationship between pore size and mass ratio increments. (j)–(l) SEM images for DLMSN with mass ratios of FCA to CTAB: (j) 0.1, (k) 0.2, (l) 0.3. Figures (g)–(l) were adapted by permission from: *ACS Appl. Mater. Interfaces*, 2020, **12**, 18823–18832.^166^

As the various and specific nature of cells indicate, they do not encompass every endocytosis route to uptake the molecules. Hence, it seems essential to control the variations and synthesis situations leading to the desired morphology and size of NPs.^165^ Different stabilizing agents should be applied to control the size and shape of GNPs. It was deduced that an increase in NP number relating to higher cellular uptake would be obtained by decreasing the size of GNPs. In the case of various morphologies of the same size, spherical amino-acid-stabilized GNPs displayed a greater increase in uptake than citrate or cetyltrimethylammonium bromide (CTAB). The strength of this work is to employ the same chemical approaches with similar stabilizing agents (CTAB) for spherical, cubic, prismatic, and rod-shaped GNPs to eschew superficial capping complexity and corona impact to compare their cellular uptake and internalization (Fig. 3a–d). CTAB was selected based on its shape-directing features, providing a situation to produce NPs in different shapes and sizes. The effects of stabilizing agents and surfactant utilization on synthesis are depicted in Fig. 3. As observed in 8 panels of prepared GNPs, the impressive impacts of size are feasible *via* a comparison of small-sized CTAB-coated spherical-shaped GNPs stabilized with citrate (Fig. 3, panel e) with larger-sized GNPs directly prepared in the presence of CTAB (Fig. 3, panel a). CTAB surfactant always acts as a shape-directing agent in the growth of metal NPs. Here, the method for producing smaller and CTAB-stabilized NPs produced initial citrate-stabilized NPs along with their superficial capping by the CTAB agent. The synthesis approach depicted in Fig. 3, panel f, is assigned to small-sized sphere-shaped GNPs synthesized utilizing a citrate reduction agent. Panel e represents GNPs with a first citrate stabilization step and a following CTAB coating step. This factor indicates that size, shape, and superficial coatings influence various GNP functions.

Dendritic large-pore mesoporous silica NPs (DLMSN) are the other category of drug carrier with high porosity. It is significant to note how the component powders were mixed or how the auxiliary template powder was added to the aqueous mixture to form micelles *via* co-assembling templates, which eventually aids in controlling the pore size and morphology of DLMSN. Here, ferrocene carboxylic acid (FCA) was utilized as an auxiliary template, and cetyltrimethylammonium bromide (CTAB) acted as a primary template. The SEM and TEM results indicated that, along with the increase in the mass ratio of FCA to CTAB (0.1 to 0.3), the DLMSN_FCA_ particle size grew from 60 to 80 nm, and the average pore entrance diameter increased from 2.8 to 12.2 nm, as shown in Fig. 3g–l.^166^

The synthesis routes as a factor to highlight in size-controlled synthesis should be discussed. There are various conventional methods for GNP preparation: *i.e.*, Turkevich and Burst. The Turkevich route was a synthesis strategy to form GNPs with a controlled size range of 1–2 nm. The central basis of this technique relies on reducing gold ions (Au^3+^) to create gold atoms (Au^0^) through reducing agents, *viz.*, amino acids, ascorbic acid, UV irradiation, and citrate.^167^ To stabilize GNPs, further capping or stabilizing agents were applied. Initially, the Turkovich approach had a limited production size for GNPs, but with the development of this method, the controlled size range for the synthesized particles reached 147–167 nm over time.^168^

The Burst approach consists of a two-phase reaction for size-controlled GNP synthesis with a size range of 1.5–5.2 nm GNPs utilizing organic solvents. First, a phase-transfer agent, such as tetraoctyl-ammonium bromide, transferred the gold salt from its aqueous medium to an organic solvent. In the next step, the reduction of gold ions occurred by utilizing a reducing agent such as sodium borohydride, followed by alkanethiol as a GNP stabilizing agent. During the reaction process, the color changed from orange to brown.^168^Table 2 presents effective parameters for the size and shape of metallic NPs.

**Table 2:** The synthesis of metallic NPs with different influential factors on their size and shape

Entry	Metallic NPs	Synthesis approach	Influential factors (stabilizing/capping agent, reducing agents, precursors concentration, temperature, pH, and time)	Shape and size	Ref.
1	Au–Pt bimetallic NPs	Concurrent reduction	P85 block copolymer, ascorbic acid, HAuCl_4_·3H_2_O (60 mM), H_2_PtCl_6_·6H_2_O (60 mM), room temperature, 50 seconds	Round/3–5 nm	169
2	Ag–Pd bimetallic NPs	Template approach	PVP, ascorbic acid, Na_2_PdCl_4_ (1 mM), syringe pump injection rate 1.2 mL h^−1^, environmental conditions	Triangular/78 nm	170
3	Pt–Au–Ru trimetallic NPs	Ultrasonic-assisted approach	PVP, ascorbic acid, RuCl_3_ (19.3 mM), HAuCl_4_ (24.3 mM) H_2_PtCl_6_ (7.7 mM), 1 h	Spherical/77 nm	171
4	Au–Ag bimetallic NPs	Concurrent reduction	CS, acetic acid, ascorbic acid HAuCl_4_·3H_2_O (0.01 M), AgNO_3_ (0.01 M), reaction applied overnight	Meso-flowers/100–1000 nm	172
5	Au–Pt–Pd trimetallic NPs	Concurrent reduction	NaBH_4_, HAuCl_4_ (1 M), H_2_PtCl_6_ (1 M), H_2_PdCl_4_ (10 mM), reaction applied overnight	Irregular/80–100 nm	173
6	Au–Pt–Ag trimetallic nanofluid	Microwave irradiation	Trisodium citrate, HAuCl_4_·3H_2_O (0.1 wt%), H_2_PtCl_6_·*x*H_2_O (0.1 wt%), AgNO_3_ (0.1 wt%), microwave heat treatment for 4 minutes	Dark nano-fluids/23 nm	174
7	Au–Ag bimetallic NPs	Laser-assisted approach	Au nano twins by pulsed laser ablation, AgNO_3_ (0.05 M), laser ablation of Au plate in distilled water	Worm-shape nano chains/6.2 nm	175
8	Pt–Au–Ag trimetallic NPs	Seed-assisted growth	PVP, citric acid, Ag seeds, H_2_PtCl_6_ (10 mM), HAuCl_4_ (10 mM), 90 °C, 3 h	Spherical/40–50 nm	176
9	Au–Ag bimetallic NPs	Seed-assisted growth	CTAB, sodium oleate, NaBH_4_, HAuCl_4_, AgNO_3_, 30 °C, 12 h	Corn-shape/100–150 nm	177
10	Ag–Au bimetallic NPs	Green-concurrent reduction	Aqueous extract of golden rod leaves, AgNO_3_ (1 mM), HAuCl_4_·*x*H_2_O (1 mM), 70–80 °C, 1 h	Spherical/15 nm	178

### 3.2. Shape controlling

Nanocarriers have been considered in drug delivery due to their unique properties, including cross-versatile biological barriers. However, this process is affected by many factors, including the nanocarrier size, morphology, concentration, and surface modification. Additionally, environmental factors, such as pH and temperature, could affect the cellular uptake and internalization procedures. In this section, we try to clarify how to control the shape of drug nanocarriers, which could directly influence their transmission across biological barriers. In addition, different noteworthy factors affecting the shape/size-controlled synthesis of metallic NPs are presented in Table 2.

**Fig. 4:**
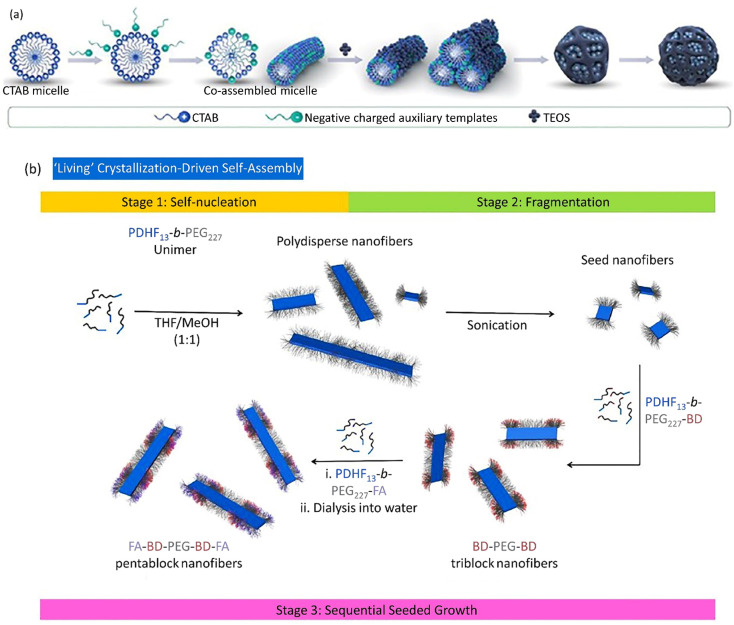
(a) The co-assembly procedure of positively charged CTAB and negatively charged auxiliary templates to form spherical DLMSN utilized a dual-templating process. Figure (a) was adapted by permission from: *ACS Appl. Mater. Interfaces*, 2020, **12**, 18823–18832.^166^ (b) A plausible preparation route of the low-dispersity PDHF_13_-*b*-PEG_227_ nanofibers produced through ‘living’ CDSA. Figure (b) was adapted by permission from: *Chem. Sci.*, 2020, **11**, 8394–8408.^179^

Spherical GNPs can be prepared through the isotropic growth of gold nuclei. Over and above that, the anisotropic growth of gold nuclei could be carried out to form diverse shapes of GNPs. The anisotropic GNPs can be synthesized *via* a two-stage seed-assisted growth procedure. First, sphere-shaped gold seeds of uniform size were produced. Second, adding gold ions with capping and reducing agents altered the reaction conditions. The produced gold seeds resemble templates on which the reduced GNPs from the second step sediment, forming large-sized GNPs with various morphologies. Since the reducing agent applied in the second stage was weak, it could simultaneously create Au^0^ from Au^3+^ in the presence of seeds and catalyze the reaction. Abundant studies have been devoted to producing various shapes of GNPs, like rod-shaped, cage-shaped, wire-shaped, plate-shaped, polyhedral, *etc.* Yang *et al.* introduced spherical DLMSN synthesized through CTAB micelle formation in triethanolamine (TEA) aqueous solution, the co-assembling of an auxiliary template, followed by the addition of tetraethyl orthosilicate (TEOS) and a hydrolysis process (Fig. 4a).^166^ A living crystallization-driven self-assembly (CDSA) seeded growth method implicated in low-dispersion 1D nanocarrier formation is based on amphiphilic block copolymer poly(dihexylfluorene)-*b*-poly(ethyleneglycol) (PDHF_13_-*b*-PEG_227_), in which PDHF acts as a core block and PEG as a corona block. This penta-block nanocarrier is arranged in a C–B–A–B–C model with 95 nm length. Since the blue fluorescence of PDHF is exposed to peripheral quenching, a far-red BODIPY (BD) fluorophore connects to the coronal B part of the terminative PEG group supplying additional explorations.

Folic acid (FA) relates to the C part as a targeting moiety. Despite many examples, few studies have utilized the living CDSA method in water media to produce low-dispersity nanofibers with controlled length to dominate the limited potential organic media for biological applications. The preparation of fragmented nanofibers should be advantageous for the existence of optimum targeting groups, *i.e.*, FA, and cargo, *i.e.*, BODIPY^630/650−*X*^, and simplified NP preparation. To overcome the complexity of the self-assembly procedure, initially, unfunctionalized PDHF_13_-*b*-PEG_227_ organized primary seed micelles. Next, PDHF_13_-*b*-PEG_227_-BD and/or PDHF_13_-*b*-PEG_227_-FA unimers were added to form fragmented nanofibers (Fig. 4b). As the endocytosis yield and optimized cellular uptake are proportional to the relation between the nanoparticle's terminating groups and the cell membrane, the nanofibers constitute PDHF_13_-*b*-PEG_227_-FA blocks.^179^

Additionally, apart from the impressive factors reported, other cellular uptake-relevant parameters are presented in Table 3.

**Table 3:** Physicochemical properties of different NPs in cellular uptake

Entry	Physicochemical property	Properties	Nanocarrier	Cell type	Uptake mechanism	Significant characteristics	Ref.
1	Size	5–100 nm	Ag	B16^a^	Clathrin-mediated endocytosis	Elevated uptake in case of larger NPs, smaller NPs pass the plasma membrane quickly	180
2	Shape	Nanospheres and nanostars	siRNA-coupled-Au	U87 glioblastoma	Endocytosis	Large spheres (50 nm) and stars (40 nm) display enhanced uptake	181
3	Corona	Hard (HC) and soft (SC) corona proteins	Hard and soft corona functionalized silica and PS	THP-1 and human brain endothelial	Endocytosis	Interactions of NPs' superficial proteins impress cell association	182
4	Superficial charge	Positive, negative and neutral	PEG-*b*-PLA^b^	Caco-2 and small intestinal epithelial	Clathrin and caveolin-mediated endocytosis	Positive charge develops cell uptake, transmission, and distribution	183
5	Hydrophobicity	Nanogels	Amphiphilic polymeric system with various hydrophobicities	Monocytic-like THP cells	Passive transmission	Polymeric network hydrophobicity influences protein attachment and cell uptake	184
6	Ligand binding	Multivalent quantum dots 15–20 nm	Galactose-modified quantum dots	HepG2^c^	Caveolae- and clathrin-interfered endocytosis	Galactose multi-valency, a major parameter in cellular uptake procedure	185
7	Mechanical characteristics	Polymer toughness	Ganglioside (GM3)-modified lipid-wrapped PLGA^d^-PLA^e^	CD169-existed on macrophages	Actin-interfered phagocytosis	Core toughness impacts NPs' cellular uptake and internalization	186

a Murine tumor cell line.

b Polylactic acid.

c Human liver cancer cell line.

d Poly(lactic-*co*-glycolic acid).

e Poly lactic acid.

The combination of low-energy mini emulsions with reversible addition–fragmentation chain-transfer (RAFT) polymerization aid in preparing convenient, fast, and eco-friendly uniform NPs. The most challenging aspect of this method is the lack of simultaneous control over the NP size, shape, and charge, so their potentiality in applications would be restricted. In the case of attaining negatively charged PS NPs, rendering various sizes from ∼100 to ∼500 nm and spherical, worm-like, and vesicle-like morphologies, only the amount of added sodium dodecyl sulfate (SDS) and toluene were adjusted.^187^

Although the reported nanoemulsion methods are restricted to producing NPs with spherical morphology, various NP shapes were prepared through the facile low-energy route for nanoemulsion production by facile shaking at an ambient temperature. This approach is based on the synergistic effect of a macromolecular chain transfer agent (macro-CTA) and SDS that diminishes interfacial tension and grants electrostatic stability.^188^

Hence, as the internalization of different NPs in versatile biological cells depends on many influential parameters, including size, shape, superficial charge and functionalization, careful monitoring of these factors during synthesis and post-synthesis processes should be considered. Furthermore, as discussed above, the stabilizing agents, pH, temperature, templates, *etc.*, are regarded as significant factors which affect the NP cellular uptake process, whose control leads to beneficial and favorable results.

## 4. Cell internalization and uptake process and influential factors

The cell membrane (CM), also known as the plasma membrane, encloses the cytoplasm by detaching the intracellular from the extracellular fluid. CM is made up of proteins contained inside a bilayer of phospholipids. Small biomolecules can enter these phospholipid bilayers courtesy of their hydrophilic heads and hydrophobic tails. More specifically, the CM is a selectively permeable barrier that regulates the entry of chemicals into the cell.^189,190^ To exchange chemicals, the CM uses a variety of mechanisms, which may be broadly grouped into passive and active transport. From areas of greater concentration to those of lower concentration, gases like oxygen and carbon dioxide, hydrophobic compounds like benzene, and uncharged molecules like water and ethanol diffuse across the membrane. Passive transport is a movement that takes place along a concentration gradient without needing energy. In contrast, active transport uses the energy that adenosine triphosphate (ATP) provides to move against the concentration gradient.^191–193^

Endocytosis is a type of active transport that allows polar or charged biomolecules that cannot pass through the hydrophobic plasma membrane to be absorbed. In this process, the cell invaginates the extracellular fluid to engulf the materials, and then the CM buds inside the cell to form an endosome, a membrane-bound vesicle.^194^ Endocytosis may essentially be divided into two groups: phagocytosis and pinocytosis. The process of taking in debris, bacteria, or other large-sized solutes by specialized mammalian cells known as phagocytes is known as phagocytosis (cell eating) (*i.e.*, monocytes, macrophages, and neutrophils).^195,196^

Opsonization, a step in the phagocytosis process, coats the target materials with opsonins such as immunoglobulins and complementary proteins to alert the phagocytes to their existence and kick-start phagocytotic activity.^197^ As the phagocyte begins to ingest the target material, it will simultaneously stimulate the formation of a membrane-bound vesicle called a phagosome into which the ingested materials are compartmentalized within the phagocyte. The hydrolytic enzymes in the lysosomal lumen degrade the materials at an acidic pH in the later stages of this process, when the phagosome and the lysosome unite.^198–200^ Pinocytosis is the process by which all cell types absorb nanoscale particles.^201^ In pinocytosis, the plasma membrane that acts as the “cellular drinking” membrane creates an invagination to absorb a tiny droplet of extracellular fluid that contains dissolved chemicals. Pinocytosis is a typical process that occurs continuously in practically all cells, regardless of the demands of the cell. The materials that have been captured are pinched off into tiny vesicles called pinosomes, which merge with lysosomes to hydrolyze or break down the contents.^202^ The size of the endocytotic vesicles is used to diagnose phagocytosis and pinocytosis; both involve the absorption of fluids through small vesicles with a size in the range of a few nanometers to hundreds of nanometers, while the former consists of the uptake of big particles by giant vesicles with a size of 250 nm.^199,203^ Pinocytosis can be divided into four different types: clathrin-mediated endocytosis, caveolae-mediated endocytosis, clathrin- and caveolae-independent endocytosis, and micropinocytosis.^204,205^ The cellular entrance mechanism used to incorporate certain compounds into cells is called clathrin-mediated endocytosis. By using the transferrin (Tf) receptor and the low-density lipoprotein receptor, this entry pathway helps cells absorb nutrients such as iron and cholesterol that are part of the plasma membrane.^206,207^ Every form of NP is taken up by the cell through a preferred uptake pathway. For instance, NPs made of silica (SiO_2_)-based nanomaterials and poly(lactic-*co*-glycolic acid), d,l-polylactide, and poly(ethylene glycol-*co*-lactide) are absorbed by clathrin-mediated endocytotic pathways.^153^ Because of their structural resemblance to the CM, the cells internalize coumarin-based solid-lipid NPs *via* a non-energy-dependent mechanism. Clathrin-mediated endocytosis is the endocytosis process used by all lipid-based NPs.^208^ Through membrane-based ErbB2 receptor-mediated endocytosis, Herceptin-coated gold NPs enter the cell.^209^ The method of cellular entrance known as caveolae-mediated endocytosis includes membrane invaginations in the shape of flasks (little caves). All cells contain caveolae, including adipocytes, muscle, fibroblasts, endothelium, and epithelial cells.^210,211^ Typically between 50 and 80 nm in size, caveolae are made of the membrane protein caveolin-1, which gives them their flask-like form.^212,213^ Endocytosis reliant on caveolae has a role in controlling membrane proteins, lipids, fatty acids, and cell signaling.^153,214^ After caveolae separate from the plasma membrane, they unite with pH-neutral cell structures termed caveosomes. Bypassing lysosomes, caveosomes protect their contents from hydrolytic enzymes and lysosomal breakdown.

Consequently, pathogens like viruses and bacteria employ this entryway to stop degradation. This pathway is used in nanomedicine since the cargo absorbed into the cells through a caveolin-dependent mechanism does not end up in the lysosome.^215,216^ Cells lacking clathrin and caveolin undergo clathrin- and caveolae-independent endocytosis. Growth hormones, extracellular fluid, glycosylphosphatidylinositol (GPI)-linked proteins, and interleukin-2 all enter cells through this pathway.^217,218^ Consider folic acid, which enters cells *via* a mechanism independent of clathrin and caveolae and is conjugated to NPs and polymers employed as imaging agents and drug delivery systems.^219,220^ A pinocytosis process known as macropinocytosis occurs when cells generate huge vesicles (0.5–10 m) known as macropinosomes to absorb vast amounts of extracellular fluid.^221,222^ Macropinocytosis is a pathway to internalize bacteria, viruses, antigen-presenting cells, and apoptotic and necrotic cells. Most other pathways cannot take micron-sized NPs into cells, but this pathway can do so. Nearly all cells, except brain microvessel endothelial cells, may undergo micropinocytosis.^223–225^

Particular molecules could apply NP surface functionalization through covalent and non-covalent bindings. The covalent interactions utilized to attach NPs to proteins, antibodies, aptamers, and peptides boost uptake in the active targeting process. In contrast, non-covalent bonds are utilized for drug loading. Comparing the reticuloendothelial system uptake of various targeted gold NRs (single-chain variable fragment (ScFv) peptide, amino-terminal fragment (ATF) peptide, and cyclic arginine–glycine–asparagine (RGD) peptide) with non-targeted NRs, it was perceived that the targeting ligands facilitated the uptake of NRs by the reticuloendothelial system. Uptake by the reticuloendothelial system tends to increase with NR ligand densities, with claims that it can be ascribed to the increased recognition of targeted NRs by the immune system in comparison with non-targeted NRs.

**Fig. 5:**
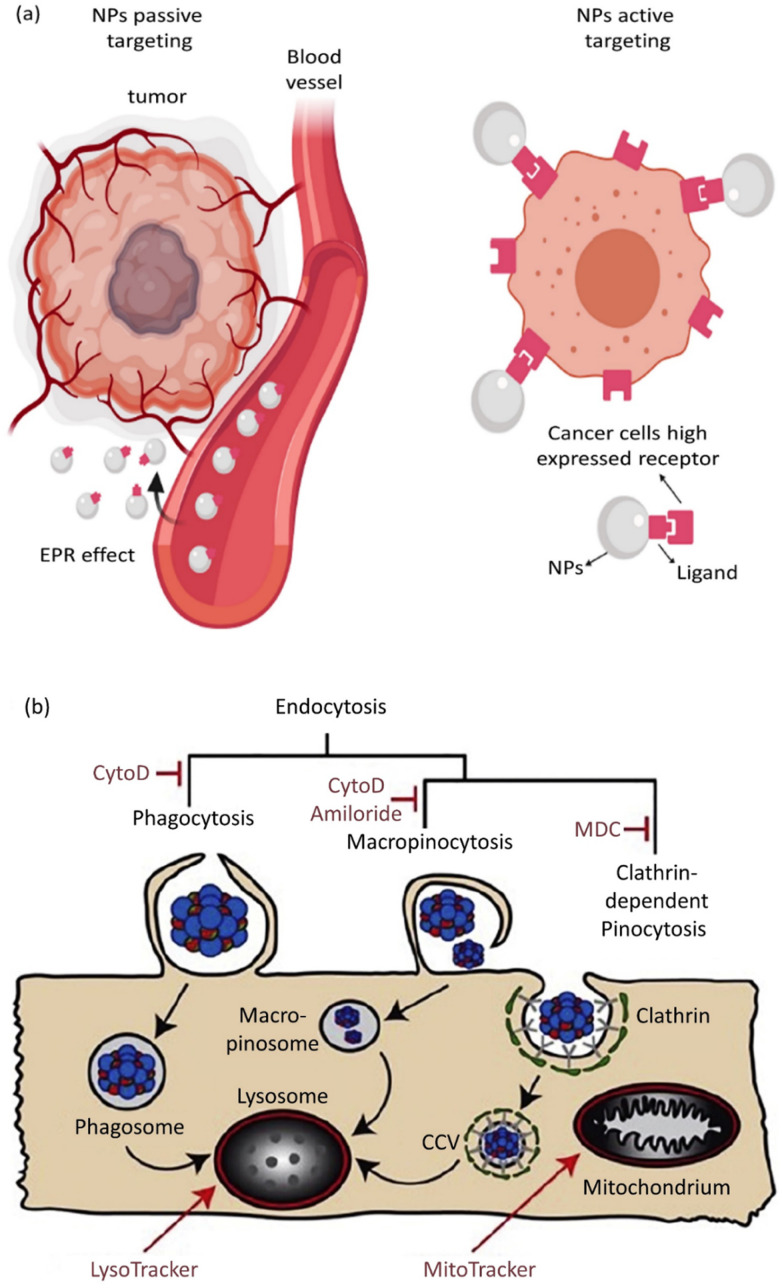
(a) Active and passive uptake of NPs. Figure (a) was adapted by permission from: *Front. Mol. Biosci.*, 2020, **7**, 381.^229^ (b) A schematic illustration of endocytic routes of the uptake of IOH-NPs into a murine alveolar macrophage cell line (MH-S cells) *in vitro*, the repressor activity, and the intracellular organelles are visible with the fluorescent tracker. Figure (b) was adapted by permission from: *J. Controlled Release*, 2020, **319**, 360–370.^232^

Based on the ability of nanoscale drug carriers to carry out accurate tasks over the last two decades, the focus on their applicability in biomedical areas, such as drug delivery and gene delivery, has been expanded. Nanocarrier aggregation in cancerous cells can mainly be conducted *via* two distinct mechanisms: passive and active targeting.^226^ In passive targeting, NPs aggregate in the tumor's vicinity due to variable penetration into tumor blood vessels. This event, known as the enhanced permeability and retention (EPR) phenomenon, allows the straightforward passive aggregation of NPs to solid tumors and/or metastatic sites *via* their specific physical features like size, morphology, and surface charge.^227^ Active targeting benefits from NP surface biofunctionalization through covalent bond attachments, which leads to selective expression by the specific receptors present on tumor cells in the tumor's microenvironment (TME).^228^ Active and passive targeting could happen simultaneously without preventing or interfering with each other's functions (Fig. 5a).^229^ The role of NPs in active drug targeting and/or contrast factors is due to the interactions between suitably surface-modified nanocarriers and molecule targets on cells and tissues. The utilized molecules for NP surface modification are classified as small proteins, peptides, antibodies, aptamers, and oligosaccharides.^230^ Moreover, NP surface biofunctionalization with mentioned particular targeting ligands is frequently demanded for reduction in toxicity (as exponentially used for SNPs) and their stability increment in biological fluids. A confirmatory example is presented by the utilization of human albumin, whose existence on NP surfaces causes a reduction in toxicity and active targeting.^231^

It is well known that NP superficial modification is applied to facilitate active targeting and cellular uptake by utilizing particular interactions between NP superficial ligands and the existing receptors on the cells.^233^ The main classification and most utilized beneficial molecules to accomplish the active uptake of NPs are antibodies, small peptides, proteins, aptamers, carbohydrates, and small molecules. The conjugation procedure, commonly covalently bonded, connects these molecules with NP surfaces to conveniently attach to the target's receptors. Monoclonal antibodies (mAbs) are applied in the active uptake of NPs based on their exclusiveness, enhanced stability, and capability of binding with the receptors. In addition, the epidermal growth factor receptor (EGFR) has been broadly applied in active targeting. As McDaid *et al.* have reported, curative m-AB-functionalized PLGA NPs attached to EGFR (Cetuximab) elevated the uptake of NPs *in vivo*.^234^ Another group of molecules that boost the specified uptake of NPs are antibodies. However, an upcoming challenge is the molecular weight of the antibodies of *ca.* 150 kDa limiting the bioconjugation procedure, especially with NPs smaller than 10 nm. On the other hand, the antibody antigen-binding fragments (Fabs) strategy is advantageous in active targeting by NPs. Paclitaxel and everolimus-loaded PEG-PLGA NPs were coated with anti-HER2 and anti-EGFR Fabs. Observation indicated that the NPs had an elevated uptake in HER2 and EGFR positive cells (SKBR3) than in negative or low EGFR-containing cells (MCF-7 and MDAMB-436).^78^ Peptides are feasible substitutions for antibodies and Fabs with strong connections to special receptors. Particular peptides are produced by selecting and sieving the phage library and separating the protein's binding chains by 3D structural analyses. Recent explorations have focused on the binding effects of natural proteins and cancer cells. Tf-functionalized doxorubicin (DOX)-loaded PLGA NPs as Tf-PLGA@DOX NPs have a high binding capability with Tf receptors (Tf-R) that exist on human HeLa epithelial cervical cancer cells and showed excellent diminished viability. Conversely, viability reduction was not desirable for immortalized HaCaT keratinocytes whose Tf-R is low.^235^ Indeed, human serum albumin (HSA) is a protein that exploits NP active drug delivery and uptake.^231^ Additionally, aptamers, tiny nucleic acid chains (dsDNA, ssDNA, or RNA), are inexpensive molecules with a convenient synthesis, which are a viable alternative to other NP superficial modification agents in boosting active uptake. The 3D structure of aptamers can attach high affinity to the receptors of cancer cells. Sgc8 aptamer-modified MSNs attached well to protein tyrosine kinase-7 (PTK-7) on human acute T lymphocyte leukemia cells. The DOX-loaded Sgc8-MSN NPs demonstrated high uptake in leukemia cells.^236^ Aptamer-modified lipid NPs loaded with all-trans retinoic acid (ATRA) were efficiently attached to the CD133 receptor of osteosarcoma cells. However, a more detailed look at the results suggests that CD133 positive cells have internalized the aptamer-functionalized NPs more than CD133 negative cells.^237^ Carbohydrates are another simple molecule involved in developing active uptake. Among screened carbohydrates, the functionalization of NPs with hyaluronic acid (HA) showed enhanced uptake *via* interacting with CD44 protein. In a recent study, HA-biofunctionalized DOX-loaded carbon dots were internalized into CD44 cells (4T1) in active targeting *via* interaction between HA-modified NPs and CD44 receptors. *In vivo* explorations authenticated the aggregation of HA-modified NPs in tumor cells.^238^ Furthermore, active-targeted gene delivery by macrophages *via* different carbohydrate-modified NPs, including mannose, galactose, and dextran, was investigated by Chen *et al.*^239^ In addition, some other small molecules are employed in the active uptake of NPs. For example, Sanitá *et al.* applied folic acid (FA)-functionalized CS–lipid hybrid NPs to elevate internalization *via* interacting with FA and folic acid receptors on cancerous cells. Interestingly, the ovarian cancer cells (SK-OV-3) had enhanced the internalization of FA-modified NPs.^240^ Additionally, anisamide (AA) and phenylboronic acid (PBA) are mentioned as other small molecules with the capability of specifically attaching sigma receptors and sialic acid (SA), respectively. AA-functionalized thymoquinone (TQ)-loaded polymeric NPs are exploited in colon cancer HT-29 with a sigma receptor: HCT-116, and Caco-2 cell lines. They revealed greater AA-TQ-NP toxicity in HT-29 cells than in HCT-116 or Caco-2 cell lines. These outcomes result from the active uptake of AA-TQ-NPs after attaching to the sigma receptor.^241^

Eukaryotic cells imply various endocytic routes for fluids, soluble molecules, and specific species. Since macrophages are the primary target cells of inorganic–organic hybrid NPs (IOH NPs), as illustrated in Fig. 5b, the uptake and internalization mechanism occurred in MH-S cells. Three heterologous pharmacological deterrents were used for this purpose: (1) cytochalasin D, (2) amiloride, and (3) mono-dansyl-cadaverine (MDC). The actin filaments are exposed to depolymerization by the first inhibitor and repress the phagocytosis and macropinocytosis mechanisms. Amiloride suppresses the Na^+^/H^+^ converter and mainly intervenes in macropinocytosis. The MDC clogs clathrin-associated pinocytosis by suppressing transglutaminase 2. The aim is to seek high efficiency and low toxicity at a defined suppressor concentration. The cellular internalized specific materials may face various destinies. Endosomes could join the lysosomes, though they end up in the mitochondria or cell nucleus or reprocess back to the membrane. Therefore, the destination of the trapped IOH NPs inside the MH-S cell was characterized by the flow cytometry method, which benefits both high efficiency and regio-selected microscopy information. As can be seen in Fig. 5b, the MH-S cells were incubated for 6 h or 24 h with betamethasone phosphate NPs (BMP NPs) comprising a green-colored fluorescent dye flavin mononucleotide (FMN) and then a red-colored fluorescent LysoTracker loaded to trace lysosomes or for inspection with the mitochondrial marker MitoTracker.^232^ Based on the high AR and deformation capability, filamentous particles have been detected in persistent circulation. Evaluating the circulation time of inert and degradable filamentous micelles showed that the circulation time of all these filamentous micelles was up to one week, 10 times longer circulation than spherical particles. The long circulation time of filamentous micelles led to elevated drug uptake by tumor cells and upgraded the dose integrated over time.

Consequently, about an eightfold enhancement in the tumor cell apoptosis number was detected than with free drugs. No difference between the circulation time of filamentous micelles with diverse flexibilities was observed. The constant circulation of filamentous particles could be assigned to the facile flow of particles throughout the bloodstream and reduced uptake by the organs of the reticuloendothelial system.

Over past decades, it has been debated whether a perfect GNP drug delivery system should afford undetectability and inertness in plasma. However, it should be activated when facing tumor site aggregation to be identified by tumor cells. Notably, several parameters have to be enhanced in the case of GNP drug carriers. The *in vivo* mechanism of various GNPs differs according to size, morphology, and other physicochemical properties. Hence, the optimization process is the most important thing to pay attention to in an efficient drug delivery process. Diverse aspects are summarized: lengthening in plasma, improvement in targeting aggregation, cellular uptake development, and drug release control inside the cells.^104^

One vital issue in nanomedicine is encapsulating theranostic agents inside NPs to lengthen the blood circulation time and to upgrade interactions with targeted cells. During circulation and based on the desired application (*viz.*, cancer drug delivery or immune modulators), NPs are required to interact with cells in human blood vessels to reduce side effects or enhance delivery efficiency. Nonetheless, evaluating the cellular interactions in blood vessels is challenging and has not been well developed or recognized because of the various components of human blood and hemodynamic flow in blood vessels. A comprehensive approach for analyzing cellular interactions of both synthetic and commercially available NPs under human blood flow conditions in a microvascular network is proposed. Significantly, this approach aids in clarifying the interactions of size, charge, and type of NPs on their cellular alliances under the dynamic flow of human blood. This approach proposes a specific platform to evaluate complicated interactions of any kind of NP under human blood flow conditions. It guides the rational design of liposomes and polymer NPs for various applications in nanomedicine.^242^ Additionally, increasing the size of carboxylic-acid-functionalized NPs enhanced cellular interaction under static conditions but lowered cellular interaction under flow conditions. Improving the size of tertiary-amine-decorated NPs leads to advanced cellular interaction under static and flow conditions. These studies confirm novel viewpoints on the associations between polymeric nanomaterials and endothelial cells.^243^

Therefore, the two main penetration routes of NPs (phagocytosis, pinocytosis, *etc.*) and mechanisms (active and passive) are intensely debated with regard to different determinations of biological molecules applied more in the active process. Additionally, NP surface functionalization enhanced the stronger linkage between the molecule and the various cell receptors in active uptake. In contrast, passive uptake depends on the accumulation of NPs near the tumor site to penetrate the cell due to the physical features of NPs like size, shape, and superficial charge.

## 5. The effect of the morphology and size of drug carriers on cellular uptake and internalization

**Fig. 6:**
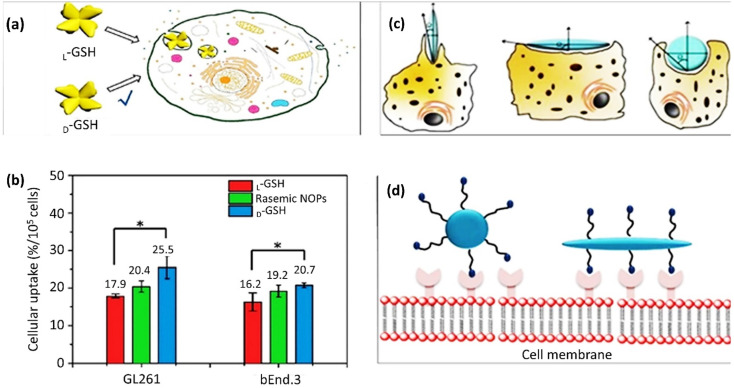
Chiral shape-dependent cellular uptake. (a) Schematic illustration of chiral shape-dependent cellular uptake. (b) Cellular uptake efficacies of chiral and racemic NOPs in GL261 and bEnd.3 cell lines during 2 h (*n* = 3). Figures (a) and (b) were adapted by permission from: *CCS Chem.*, 2021, 773–783.^244^ (c) The effect of NP morphology on cellular uptake; (d) the interactions between spherical and rod NPs cell overexpressed receptors *via* active targeting. Figures (c) and (d) were adapted by permission from: *Mater. Horiz.*, 2020, **7**, 1727–1758.^247^

NP size is an essential factor with a massive impact on cell membrane transportation. GNPs with a broad size spectrum from thousands of nm could pass through the cell membrane by endocytosis. However, this transmission strategy is not practicable for smaller NPs. A recent study has reported enantiomorphic gold nanooctopods (NOPs) with chiral morphology synthesized *via* a seed-mediated approach to evaluate the morphology effect on cellular uptake. These chiral NOPs presented two opposite l-glutathione (l-GSH) and d-glutathione (_D_-GSH) handedness, leading to contrary circular dichroism signals. Improved cellular uptakes of 40% and 30% were obtained by d-GSH NOPs in mouse glioblastoma (GL261) and mouse brain endothelial (bEnd.3) cell lines, respectively, compared to racemic NOPs, including l-GSH (Fig. 6a and b). The chiral-shaped dependency of cellular uptake could be assigned to the preference for PEG-functionalized d-GSH NOPs or l-GSH. According to Fig. 6b, the cellular uptake of racemic NOPs was between l-GSH and d-GSH NOP contents.^244^ Additionally, the modulation of cellular uptake is affected by the NP morphology. Versatile synthesis and modification methods have been exploited to produce NPs with different morphologies, including nanoscale triangles, rods, spheres, stars, and cubes. Among them, rod-shaped GNPs displayed elevated membrane adhesion efficacy compared to spherical GNPs.^245^ In a comparative study on the internalization of GNPs by the various rod, cube, sphere, and prism morphologies, the sequence of nanocarriers entering the membrane was: rod > cubic > spherical > prism-like GNPs.^165^ The orientation of non-spherical NPs, such as rod-shaped NPs, in connection with the receptors on the cell membrane, influences the cellular uptake and internalization (Fig. 6c). The development was announced of the internalization content counted by the number of internalized NPs per cell in the case of a tangent angle (*Ω*) less than 45°. This amount is smaller than the value for spherical NPs. The internalization limitation occurs at *Ω* > 45° since the NPs can only distribute at the cell membrane but cannot be taken up by cells. The entrance mechanism of NPs with *Ω* = 90° suggests that the internalization of perpendicular shape-stable particles resembling cellulose nanomaterials would be beneficial. Accordingly, hard non-spherical NPs demonstrated higher cellular uptake compared to soft NPs.^246^ Non-spherical NPs have particular ligands to fit the membrane receptors in an active targeting mechanism, which could enhance receptor-based endocytosis because non-spherical shapes, such as rod NPs, share their abundant binding sites with cell membrane receptors (Fig. 6d).

Furthermore, sharp-shaped NPs have an increased propensity to permeate the endosomal membrane and have advanced cytoplasmic localization restricting the possibility of NPs being expelled through exocytosis.^247^

The cellular uptake and internalization procedure for GNPs are influenced by their size, shape, surface charge, surface functionalization, and the relations between these factors. Therefore, to achieve a desirable cellular uptake efficiency, considering these elements in every step is the point to be highlighted.^104^ Resizing is the principal action among other influential agents mentioned. Nevertheless, there is no agreement on size optimization and its impact on cellular uptake. For example, Barui *et al.* indicated the size-dependent biomedical functions and cellular uptake of GNPs in osteosarcoma cells. The synthesized NPs were in a 40–60 nm size range. In conclusion, 46–60 nm GNPs had a higher cancer cell elimination rate than 38 nm GNPs at concentrations of 200, 400, and 800 ng mL^−1^.^248^

In contrast, Engstrom *et al.* proved that more minor lipid bilayer-coated GNPs, *i.e.*, 5 nm and 10 nm, can internalize a lipid monolayer with high convenience and low energetic costs due to the sum-frequency generation (SFG) technique. In comparison, 20 nm GNPs warped the membrane and caused a curved form of hybrid lipid-coated GNPs.^249^ Shape modification is the next significant, influential factor in cellular uptake. Nanospheres, nanorods, nanostars, nanocages, nanoshells, *etc.*, are examples of the most familiar morphologies of GNPs. Among them, nanorod GNPs are the most attractive and efficient shapes according to their small cross-section, leading to rapid intracellular internalization.^86^ Significantly, nanorod GNPs rather than nanospheres implied the development of fluorescence. This feature resulted from the LSPR adaptability of nanorod GNPs, which coordinates with the spectrum of red/NIR dye. It causes an impressive enhancement in the fluorescence of red and NIR dyes, achieving optimized fluorescence recovery.^88^ Additionally, rod-shaped GNPs exhibited boosted biological functions compared to spherical GNPs because of their unique optical characteristics and changeable and AR-dependent plasmon bands. To show the importance of the AR of rod-shaped GNPs on the internalization rate in green algae *Raphidocelis subcaptata*, Zucolotto *et al.* synthesized rod-shaped GNPs with 1.90, 2.35, 3.25, and 3.50 ARs at concentrations of 2 and 10 μg mL^−1^. Better efficiencies were detected in the incubation of algae with GNPs with the highest AR (3.50).^250^ Other influential factors in the internalization process include the superficial modification of NPs with different species and the superficial charge of nanocarriers in cellular uptake, some of which are mentioned in the following. The PEGylation of NPs increases their cellular uptake and stability in biological fluids along with a reduction in accumulation due to the properties of the PEG monomer, such as small size (0.35 nm length), 2 kDa molecular weight, and length tunability. The PEG-functionalized NPs have reduced interactions with un-specified proteins leading to the “stealth” effect, which improves the prolonged blood circulation of PEG-functionalized NPs and diminishes phagocytosis. The more stable the PEG-functionalized NPs, the greater the cellular uptake compared with bare NPs whose fate is accumulation in biological surroundings and/or exposure to phagocytosis by immune system cells.^251^ PEGylated particles with the lowest AR achieved the highest accumulation in the tumor site. Nevertheless, this outcome may represent the pore size impact of the leaky vasculature, which means that smaller NRs have the chance to leave the systematic circulation and accumulate in cancerous tissue. The tumor homing of peptide-coated NRs has passive and active targeting strategies, with the mid-sized NRs exhibiting the advantageous integration of these two strategies. Moreover, PEGylated NRs demonstrated advanced tumor accumulation compared to peptide-coated NRs with the same AR. This is ascribed to the effective circulation time of PEGylated particles. Hence, the propensity of NRs to maximize the amount of drug accumulated in the tumor, the circulation time and transport from the circulation system to tumor tissue should be evaluated. Moreover, the anti-PEG antibodies (anti-PEG immunoglobulin M (IgM)) and an immunological response can be produced in response to the administration of PEGylated medicine.^252^ Due to these occurrences, the biological advantage of PEG-conjugating medicines or NPs frequently only last for the first dosage of a therapy term. By the second dosage, the mononuclear phagocyte system in the spleen and liver has identified the PEGylated drugs, and they are swiftly removed from circulation.^253^ It has been found that anti-PEG IgM is a significant marker of and contributor to the ABC of PEGylated nanomaterials. According to several clinical trials, anti-PEG IgM can also be found in healthy people who have never taken medicine that has been PEGylated.^254,255^ As a result, it has been shown that the influence of the anti-PEG IgM-mediated clearance of PEGylated NPs presents a growing problem for developing PEG-associated therapeutic techniques. Due to the physical interaction of PEGylated graphene oxide (nGO-PEG) with the macrophage membrane, which enhanced cell mobility and migration, Luo *et al.* confirmed that nGO-PEG generated robust immunological responses.^256^ The result was that the activated macrophages produced activation-associated cytokines at a greatly accelerated rate, including interleukin-6, monocyte chemotactic protein-1, tumor necrosis factor, interferon, and interleukin-12, with cytokine levels reportedly directly proportional to nGO-PEG dosages. Kupffer cells have also been found to contribute to PEG-related ABC by Lai *et al.* Antigen-presenting cells (APCs), known as Kupffer cells, which link the innate and adaptive immune systems, have been demonstrated to remove PEGylated liposomes from the body after being recognized by anti-PEG antibodies.^257^ The term “accelerated blood clearance” (ABC) refers to this unanticipated immunogenic response that causes fast clearance and reduced therapeutic agent effectiveness.

Additionally, the modification of NPs with some structures and molecules leads to alteration in the NP surface charge. For instance, amine-terminated molecules (R–NH_2_) at physiological pH (7.4) have a positive charge.^258^ Moreover, the NP surface charge could be favorably altered for tunable cellular uptake. Zwitterionic ligands, including carboxybetaines and sulfobetaines, exhibit a large group of positive and negative species permitting the modifications of charge densities for solubility optimization and eschewing the interaction of protein corona to justify the increased solubility of NPs in biological fluids. This process decreases non-targeted cell uptake and upgrades the aggregation of modified-NPs in target cells. For example, Drijvers *et al.* have biofunctionalized silica-coated CdSe/CdS quantum dots with PEG and once with sulfobetaine to investigate the effects of these NPs on cellular uptake and internalization. They deduced that sulfobetaine zwitterion-modified NPs were taken up into the target HeLa cells more conveniently than PEG-modified NPs.^259^

Interestingly, the decreased uptake of NPs caused by a negatively charged dye, pyranine, could be reversed by positive molecular cage addition, which neutralized the previous dominant-negative surface charge.^260^ Another study revealed that incremented internalization was proportional to positively charged NPs.^261^ Notably, cell-penetrating peptides (CPPs) modify the NPs to increase cellular uptake. These modifying molecules consist of a particular amino acid succession, generally polycationic or amphipathic structures, to upgrade the uptake of NP cells. The Tat peptide-superficial functionalized poly(lactic-*co*-glycolic acid) as PLGA NPs can easily penetrate and internalize the HeLa cells and increase the cellular uptake compared to bare NPs.^262^ The chain length of modifying molecules could also impact the internalization of NPs. Modified phosphatidylcholine (PC) with various alkyl chain lengths (from C12 to C18) was proportional to the increase in internalization of lipid–PLGA hybrid NPs.^263^Table 4 represents some nanoparticulate systems for drug delivery with successful cellular internalization procedures in cancer treatment.

**Table 4:** The nanoparticulate systems as enhanced drug vehicles for cancerous tumor therapy

Entry	Nano vehicle	Composition	Loaded substance	Reported results	Ref.
1	Polymeric NPs	Polydopamine, alendronate	Paclitaxel	Incremented aggregation in tumor cells than other tissues	264
2	Liposomes	DSPE-mPEG^a^, cholesterol	DOX and SiRNA	The incremented nuclear concentration of liposomes, double OSA^b^ cells' displayed by surface EphA2^c^ receptors and JIP1^d^ proteins inside the cells	265
3	GNPs^e^	Tannic acid, HAuCl_4_	—	Incremented presence of proapoptotic protein Bax in the cancer cells and diminished presence of anti-apoptotic protein Bcl-2^f^	266
4	Metallic NPs	Fe^3+^ ions self-assembly with anchored HA	Zoledronate	Osteoclast activity's prohibition, killing cancerous cells with produced free radicals	267
5	Zinc oxide NPs	Titanium substrate and zinc acetate	Naringin	Reproduction of large bony deficiencies in OSA, bacterial RNA, and DNA leaking after ROS^g^ aggregation in the cells	268
6	Gold-aryl NPs	C_6_H_4_-4-COOH link in gold	Bovine serum albumin	Uptake and internalization in cancer cells	269
7	MSNs^h^	Polyacrylic acid, lectin	DOX	8-times larger cytotoxicity compared to free drugs	270
8	Micelles	PEG, polyurethane	DOX	Remarkable antitumor activity *versus* Saos-2^i^ cells	271
9	Micelles	Polypeptide (methoxy poly(ethylene glycol)-*block*-poly(*S-tert*-butylmercapto-l-cysteine) copolymers)	DOX	Reduced aggregation in the heart and elevated aggregation in cancer cells, metastasis deterrence	272
10	MNPs^j^	Polyethylenimine, dextran, iron oxide	miR-302b^k^	Magnetic field-responsive NPs in cancer cells displayed cytotoxic impact	273
11	NPs-loaded photoactive mesenchymal stromal cells	Polymethyl methacrylate	Human osteosarcoma MG-63 cells	Photodynamic-driven cancer cells elimination	274

a Distearoyl phosphoethanolamine.

b Osteosarcoma.

c Ephrin alpha 2 receptor.

d JNK-interacting protein 1.

e Gold nanoparticles.

f B-cell lymphoma 2.

g Reactive oxygen species.

h Mesoporous silica nanoparticles.

i Osteosarcoma cell line.

j Magnetic nanoparticles.

k MicroRNA 302b.

The evaluation of the effect of surface functional groups often includes PEG with high hydrophilicity, the negative carboxyl group (–COOH), neutral functional groups like hydroxyl groups (–OH), and the positive amine group (–NH_2_). Increasing the functional amine groups increases the positive surface charge leading to an increase in NP uptake into cells for diverse cell lines, *i.e.*, phagocytic and nonphagocytic, and for different NPs. Carboxyl (–COOH) functional groups endow the NPs with a negatively charged surface and demonstrate an increase in NP uptake in both cell types. Investigations on uptake were operated in a protein-containing medium (fetal calf serum). This would develop particle uptake. However, surface carboxyl groups of dendrimers caused increased residence time *in vivo*. A plausible description is resistance to recognition by the immune system *via* protein adsorption. These contrary results could be ascribed to other NP properties, namely the hydrophilicity or diversity in experimental conditions that affect the NP uptake and differ between different experiments (*in vitro vs. in vivo*).^275^ PEG-coated NPs diminish particle uptake and clearance by immune cells, which increases the circulation times of NP therapeutics. However, based on technical barriers in quantifying the amount of PEG grafted and generating dense PEG coatings, no extensive studies have meticulously evaluated the PEG grafting density which is essential to attain desirable “stealth” characteristics. The “grafting to” strategies based on covalent conjugation facilitates higher PEG grafting density in comparison with other mechanisms, including postinsertion, adsorption, or phase separation. These strategies can also lead to sufficient grafting density to readily resist uptake *via* immune cells.

In line with this, polymeric NPs with precisely adjustable PEG grafting were applied. It was claimed that, for a wide range of PEG lengths from 0.6 to 20 kDa, PEG coatings at density are significantly greater than those demanding brush-like PEG conformation that is exceptionally resistant to uptake by cultured human macrophages and primary peripheral blood leukocytes. Less than 20% of these NPs were cleared from the blood after 2 h (*t*_1/2_ ∼ 14 h) in BALB/c mice, while less densely PEGylated and uncoated control particles were virtually restricted within 2 h. According to the results, the stealth characteristics of PEG-coated NPs are dependent on obtaining PEG grafting at densities exceeding those needed for brush conformation.^276^ Cell features have been shown to affect the cellular uptake of NPs. Consequently, uptake is related to differences in cell type, *i.e.*, phagocytic *vs.* nonphagocytic, cancer *vs.* normal cells, and monocytes *vs.* macrophages. Cancer cells have been demonstrated to represent various amounts of surface receptor compared to normal cells. This has a huge impact on the availability of cargo binding sites and their uptake. Moreover, the metabolic activity of cells utilized ought to affect NP uptake, as has been depicted with contradictory results. As expected, small differences in uptake kinetics and amount of NPs taken up are detected across species.^277^ Phagocytes and nonphagocytes have similar biological functions across all species; thus, the detected differences between the similar endocytosis-type cells are small, and the trend in uptake is analogous. The discrepancies can be attributed to the small variations in the composition of the cell membrane, the available surface area, and the cell volume or size.

Following accurate considerations of the various morphologies of NPs and different ways to control their size and shape, it seemed vital in this section to discuss the impact of the size and shape of NPs on cellular uptake and internalization. As a result, the proportional relations between shape- and size-controlled NPs and internalization were confirmed. Moreover, the cancerous cells effectively took up the size-controlled drug carriers and enhanced the therapeutic efficiency. Additionally, the distinctive optical features of the rod-shaped GNPs are preferred to the spherical carriers.

## 6. The evaluation methods of cellular uptake and internalization

Imaging techniques in biomedical fields have progressed quickly over the last two decades, particularly in cancer tumor therapy, to distinguish even microscopic cancer piles. This imaging progression consists of combining classical with innovative imaging techniques.^278^ Generally, the methods used for evaluating the internalization of NPs are divided into label-free and label-based approaches. Transmission electron microscopy (TEM), scanning electron microscopy (SEM), and Raman microscopy are some examples of label-free techniques.^279^ There are no requirements for fluorophore usage in the label-free method, which affects the size and chemical features of NPs. Moreover, the utilization of labeled-NPs complicates differentiation between uptake and internalization of NPs to the cell and NPs attached to the cell membrane. The exerted fluorescent signal emitted from NPs is the original basis of label-based techniques. This signal could be assigned to the inborn characteristics of NPs or the combination of a fluorophore with an NP.^280^

### 6.1. Confocal microscopy

**Fig. 7:**
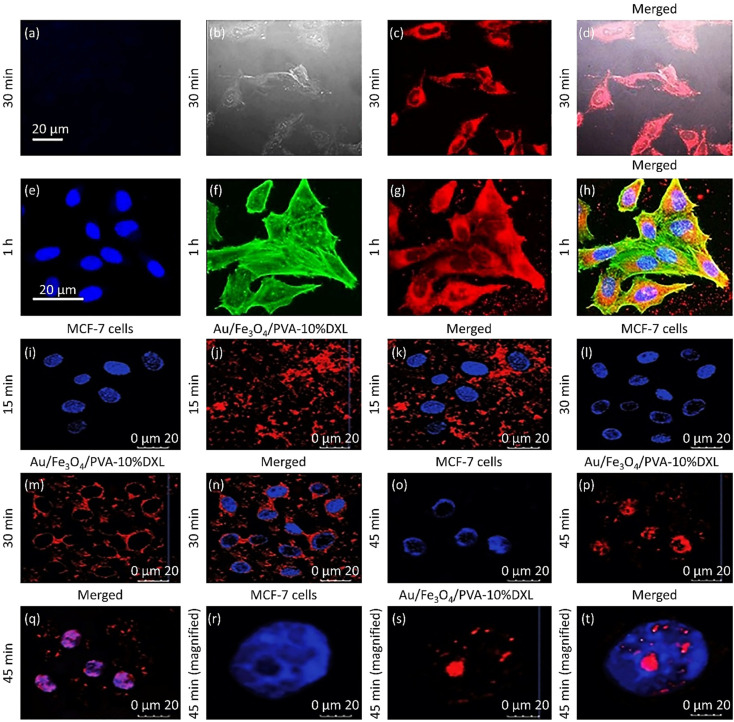
All of the scale bars of the (a)–(h) images are in accordance with 20 μm. (a)–(d) Confocal laser scanning microscopy (CLSM) maximum intensity projections of live HeLa cells subject to FA-BD-PEG-BD-FA penta-block nanofibers after 30 min (10 μg mL^−1^, *L*_n_ = 95 nm, *L*_w_/*L*_n_ = 1.17, *σ*_L_ = 39 nm): (a) the PDHF core marked as blue channels (*λ*_ex_ = 405 nm, *λ*_em_ = 415–478 nm); (b) the brightfield transmitted channel; (c) red channel obtained from BD fluorescence (*λ*_ex_ = 633 nm, *λ*_em_ = 640–700 nm); (d) overlaid images of (a)–(c). (e)–(h) CLSM maximum intensity projections of immobilized HeLa cells subject to dual-emissive FA-BD-PEG-BD-FA penta-block nanofibers after 1 h (50 μg mL^−1^): (e) nucleus blotted with DAPI; (f) F-Actin blotted with Alexa Fluor 488 Phalloidin; (g) BD fluorescence emitted from FA-BD-PEG-BD-FA penta-block nanofibers; (h) overlaid images of (e)–(g). Figures (a)–(h) were adapted by permission from: Chem. Sci., 2020, **11**, 8394–8408.^179^ (i)–(t) A set of confocal microscopy images MCF-7 cells (blotted with crystal violet) and 45 min incubation with Au/Fe3O4/PVA-10%DXL (product 7) demonstrate internalization of cellular uptake. Figures (i)–(t) were adapted by permission from: *Small*, 2020, **16**, 2002733.^8^

Thanks to the to increased resolution, image contrast, and penetration depth, even low quantities of NPs and NPs centralized to cellular sections can be detected with the aid of confocal fluorescent microscopy (CFM).^281^ To figure out how inclusion of folic acid (FA) could influence the facile cell uptake and internalization (10 μg mL^−1^, *L*_n_ = 95 nm, *L*_w_/*L*_n_ = 1.17, *σ*_L_ = 39 nm) of dual-emissive PDHF_13_-*b*-PEG_227_ segmented nanofibers, they underwent a 30 min incubation with HeLa cancerous cells and were imaged by confocal laser scanning microscopy (CLSM). After this incubation, a remarkable cellular uptake of FA-BD-PEG-BD-FA nanocarriers was perceived (Fig. 7a–d). Despite no fluorescence being detected in the blue channel for PDHF, considerable fluorescence was detected from BD. Pointwise fluorescence emerged within the cell all over the cytosol, focused around the nuclear area.

In contrast, very few nanofibers were detected to have been taken up in the central area, likely the nucleus. Fluorescence detected on every side of the perinuclear area was possibly related to nanofibers settled around the nuclear membrane. Additional CLSM explorations were conducted with the DAPI-labelled cell nucleus and Alexa Fluor 488-Phalloidin-labelled F-actin (Fig. 7e–h). They authenticated low-intensity fluorescence detected within the nucleus, which alluded to the inability of the nanofibers to concentrate within the nucleus. The *z*-stack data evaluation for fixed and live cells disclosed that pointwise fluorescence was concentrated in the cell rather than the surface, confirming internalization of the nanofibers within the cell, and corroborating no attachment of nanofibers to the cell membrane.^179^

In a recent study, Maleki *et al.* designed an efficient docetaxel (DXL)-loaded nanocomposite (Au/Fe_3_O_4_/PVA-10%DXL) with controlled release smart delivery in breast cancer-targeted treatment. CFM images were implemented with crystal violet stained cells to verify the internalization and uptake into MCF-7 breast cancer cells. Crystal violet is a material with maximum photoluminescence emission at ≈600 nm, higher than DXL emission. After 30 min of incubation, Fig. 7i–t distinctively exhibit the co-localization of Au/Fe_3_O_4_/PVA-10%DXL with the MCF-7 cancerous cells and the nanocomposite brought to the cell surfaces as well. Over the additional incubation time (45 min), the nanocomposite has been excellently taken up by the cell, which is corroborated by the high contrast between blue-stained MCF-7 cells and the red-colored Au/Fe_3_O_4_/PVA-10%DXL nanocomposite. After 45 min incubation, the major concentration of DXL in the cell nuclei and cytoplasm vesicles is depicted in Fig. 7t.^8^ Another study focused on QDs covalently attached to folic acid (FA) for explorations of the cellular uptake of FA receptors in breast cancer cell treatment, utilizing the HeLa cell line. CFM used this conjugate to examine FA receptors in HeLa, MCF7, MDA-MB231, and T47D cells. The results indicated that FA cellular uptake was rapid, and 30 min incubation was sufficient for receptor endocytosis.^282^ In addition, decoding the cellular uptake and internalization mechanisms of a particular gene silencing system comprising WRAP:siRNA was visualized by CFM.^283^

### 6.2. Flow cytometry

The flow cytometry approach is broadly utilized to monitor physiological procedures, such as the cellular uptake of NPs, and it could show the internalization routes for NPs. However, it is impossible to distinguish the NPs attached on the cell membrane from the cell-internalized NPs.^284^ In a recent study directed by Fixler *et al.*, approaches to distinguish M1 and M2 macrophages both *in vitro* and *in vivo* aid human health status forecasts. The phagocytic character of macrophages and scattering properties of label-free rod-shaped GNPs synergistically overcome this issue. The internalized rod-shaped GNPs, relating to their consumption amount, afford a noticeable scattering profile at the red channel in the flow cytometry method, reducing the cellular side scattering. Only the surface chemistry of the rod-shaped GNPs controls the internalization. Poly(allylamine hydrochloride)-rod GNPs (PAH-rod GNPs) exhibited the highest intake potential pursued with sodium citrate (Cit-rod GNPs), PS sulfonate (PSS-rod GNPs), and polyethylene glycol (PEG-rod GNPs). Importantly, PAH-rod GNPs cause dissimilar uptake between M1 and M2 cells, showing 3 times higher M1 intake than M2.^285^

**Fig. 8:**
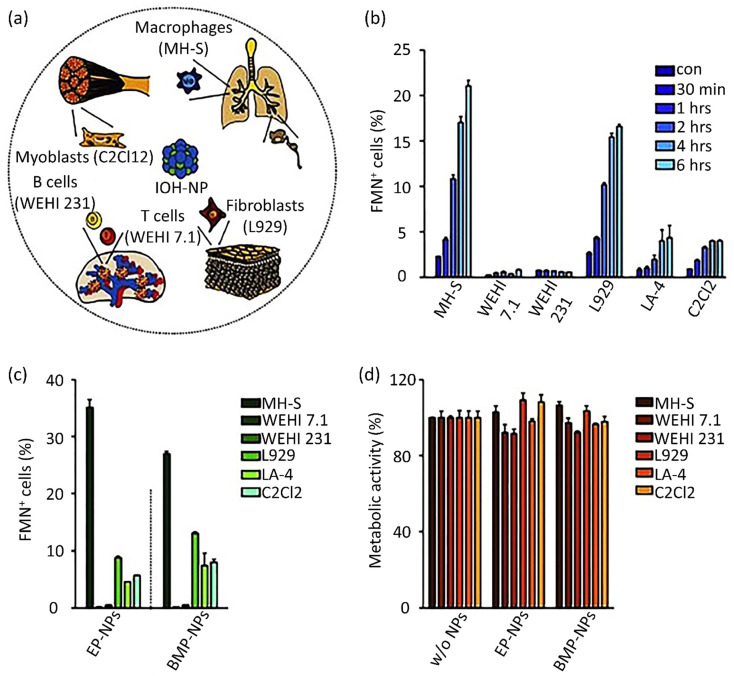
The selectivity of different cell kinds *in vivo* IOH-NP uptake: (a) a schematic illustration of the basis for the employed cell lines; (b) the cell line culturing in 6-well plates and 6 h of incubation with 2.5 μg mL^−1^ EP NPs including FMN. IOH-NP-free cells were analyzed as the control group (con). The cellular uptake efficiency was conducted due to FMN^+^ cell percentage (*N* = 3) with a flow cytometry method: (c) 24 h incubation of cell lines with 2.5 μg mL^−1^ EP NPs or BMP NPs was analyzed based on FMN^+^ cell percentage (*N* = 3) with the flow cytometry method; (d) 6 h incubation of cell lines with or without 2.5 μg ml^−1^ EP-NPs or BMP-NPs where an MTS method was used to assess their viability based on their metabolic activity (*N* = 4). Cells cultured without (w/o) IOH-NPs served as a reference. This figure was adapted by permission from: *J. Controlled Release*, 2020, **319**, 360–370.^232^

The activity of nanocarriers is estimated by distinctive ability of specific cells to uptake special species through endocytic routes. Under this method, as shown in Fig. 8a, six cell lines originating from the immune system, and even solid tissues were chosen. The uptake efficiencies of these cell types were accomplished with IOH NPs by flow cytometry of FMN contained in BMP NPs and also [ZrO]^2+^[(HPO_4_)_0.9_(FMN)_0.1_]^2−^ as EP NPs. The obtained data agreed well with the confocal microscopy results for MH-S cells. First, each cell line was incubated for 6 h with EP NPs and the cell percentage that had combined with them during this time was compared. The MH-S cells emerged with efficient uptake, while the WEHI 7.1 and WEHI 231 cells exhibited negligible internalization (Fig. 8b). As depicted in Fig. 8b, L929 cells interconnected with EP NPs very quickly, whereas LA-4 and C2Cl2 cells showed mediocre uptake efficacy. In the next level, each cell line was incubated for 24 h with EP NPs or BMP NPs. The longer the incubation time, the higher the percentage and uptake of FMN^+^ cells. However, the uptake efficiency order of IOH NPs for each cell remained identical (Fig. 8c). Eventually, as shown in Fig. 8d, the metabolic activity measured by the MTS method§§The MT assay is utilized to evaluate cell proliferation, cell viability, and cytotoxicity. confirms that IOH NPs do not influence cellular viability. In concert, the obtained results indicated that preferential targeting for selected different cell kinds and tissues using the cellular uptake and internalization of IOH NPs is feasible.^232^

Another study focused on the cellular uptake efficiency of a low-dispersive 1D nanocarrier built up from poly(dihexylfluorene)-*b*-poly(ethylene glycol) (PDHF_13_-*b*-PEG_227_) block copolymer. PDHF forms the core part of the blocks, and PEG forms the corona part of blocks containing various terminative functional groups. This 1D penta-block nanofiber with a C–B–A–B–C arrangement was synthesized with a length of 95 nm. Since PDHF blue fluorescence is frequently exposed to peripheral quenching, a far-red BD fluorophore was connected to the terminating group of PEG in the B part to explore this possibility further. Folic acid (FA) was also connected to the terminative C part as a targeting agent. Hence, the as-prepared dual-emissive penta-block nanocarrier demonstrated more than 97% of the uptake of folate receptor-positive HeLa cells measured by flow cytometry. The FA-free nanocarriers did not display any remarkable uptake (less than 1%). The lower cellular uptake in the case of FA-free nanocarriers provides a hint for the smart and targeted diagnosis and preferential internalization of folate-receptor-containing cells. Additionally, no considerable toxicity was detected for FA- or BD-containing nanocarriers.^179^

### 6.3. TEM

Transmission electron microscopy (TEM) is a label-free method presenting high-resolution images up to the cell scale, though it is costly and takes time. TEM is a destructive imaging approach, unlike Raman microscopy. This method has broad use in investigating the uptake and internalization of nanocarriers.^286^ In a recent study, aggregations of GNPs with a glutathione corona coating, functionalized with a dansyl chromophore (a-DG-AuNPs), were prepared and acted as an effective nanocarrier in photothermal therapy (PTT). The NP accumulation upgrades the radiative excitation quenching and subsequent changes into heat. The a-DG-AuNPs can internalize human hepatocytes Hep G2 cells.

**Fig. 9:**
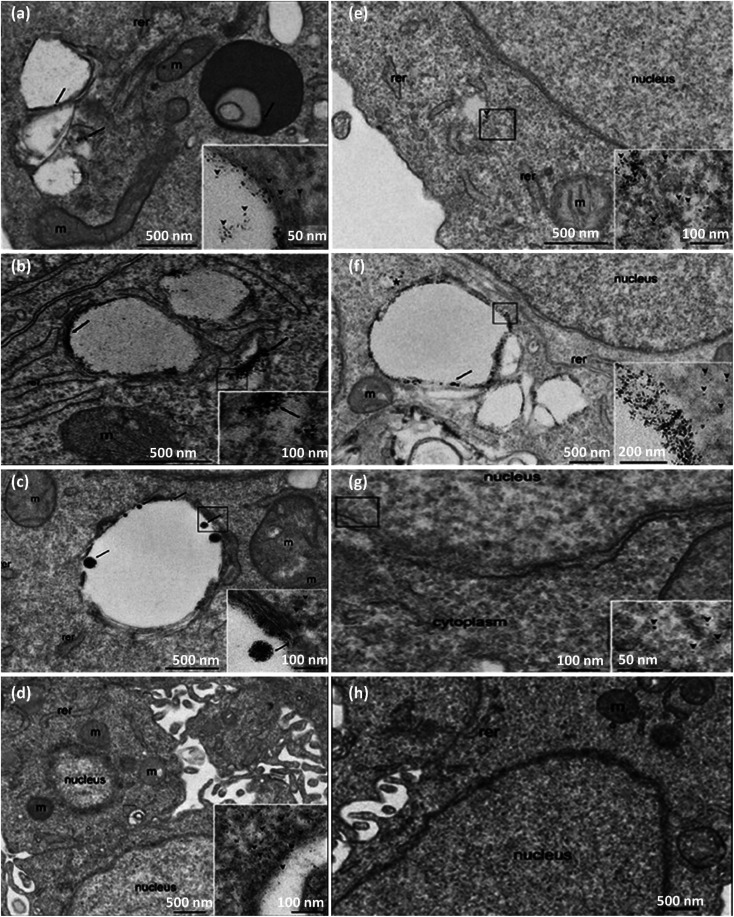
TEM images of Hep G2 cells incorporating aqueous suspensions of: a-G-GNPs, concentration: 70 μg mL^−1^, duration: (a) 1 h or (b) 2 h; a-DG-GNPs, concentration: 70 μg mL^−1^, duration: (c) 1 h or (d) 2 h; (e) a-G-GNPs or (f) a-DG-GNPs both with the same 700 μg mL^−1^ concentration and 1 h duration. (g) Magnification of the Hep G2 cells incorporated with a-G-GNPs (70 μg mL^−1^, 1 h) provides proof of the uptake and internalization of GNPs into the nucleus (the black arrowheads in panel (g)). (h) Untreated Hep G2 cells as control examination. (m = mitochondria; rer = rough endoplasmic reticulum; n = nuclelus). This figure was adapted by permission from: *Biochim. Biophys. Acta, Biomembr.*, 2020, **1862**, 183252.^283^

The Hep G2 cell cultivations were placed into plates and treated for 1 h and 2 h with 1.0 mL of 70 μg mL^−1^ or 700 μg mL^−1^ of a-G-GNPs or a-DG-GNPs suspensions with 80–90% cellular convergence. The TEM micrographs of 1 h exposure of the cells with a-G-GNPs (70 μg mL^−1^) display accumulations of ∼50 nm (indicated with black arrows in Fig. 9a) taken up into vesicles, the same as many single NPs found in the cytoplasm, organelles, and nucleus (indicated with black arrowheads in Fig. 9a and g). After incubating for 2 h, the accumulations were found to be in some vesicle-like structures, and the cytosol (indicated with black arrows in Fig. 9b) along with individual NPs (indicated with black arrowheads in Fig. 9b). Exposure of the cells with a-DG-GNPs (70 μg mL^−1^) for 1 h produces large accumulations of about 50–100 nm inside vesicles (indicated with black arrows in Fig. 9c). In contrast, many individual NPs are placed in the cytoplasm (indicated with black arrows in Fig. 9e). After incubation for 2 h, higher values of insulated GNPs are found in the cytoplasm (indicated with black arrows in Fig. 9d). At a concentration of 700 μg mL^−1^, some vesicles with aggregates and insulated GNPs are detected in the cytoplasm (Fig. 9e and f). Internalized large NP accumulations were detected at a concentration of 700 μg mL^−1^ of a-DG-GNPs (Fig. 9f). These results indicate that the internalization procedure happens through invagination of accumulation-containing vesicles. This procedure does not flawlessly influence the cell membrane. Notably, insulated GNPs capable of nucleus permeation were released when subject to the intracellular environment (Fig. 9g).^283^

### 6.4. Raman microscopy

**Fig. 10:**
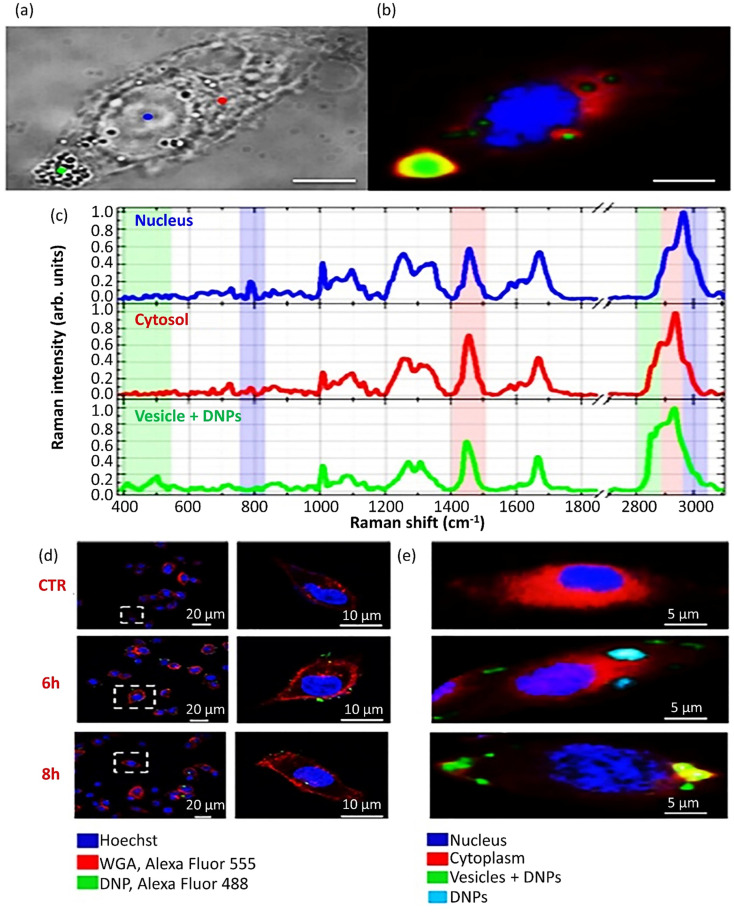
Raman microscopy of diatomite-based NPs uptake in an epidermoid lung carcinoma cancer cell line (H1355). (a) An optical image of H1355 cells after incubation for 24 h with diatomite-based NPs. The observed dots are correlated to the nucleus, cytoplasm, and vesicles represented in Raman spectra. (b) Reconstruction of the Raman image of the chosen cell by the MCR approach. The blue, red, and green colors represent the nucleus, cytoplasm, and lipid vesicles incorporated with diatomite-based NPs, respectively. (c) Representations of Raman spectra for the nucleus, cytoplasm, and lipid vesicles with diatomite-based NPs are depicted in blue, red, and green, respectively. The uptake kinetics of diatomite-based NPs in H1355 cancerous cells. (d) Confocal microscopy. (e) Raman imaging. These results were obtained after 0, 6, and 18 h of incubation. This figure was adapted by permission from: Journal of Biophotonics, 2018, **11**, e201700207.^288^

Raman microscopy needs less sample preparation and provides the possibility of *in vitro* and *in vivo* imaging of cells classified in label-free techniques. Therefore, it is considered a beneficial technique for pursuing the uptake of nanocarriers, among other label-free methods.^287^ This approach was applied in Managò *et al.*‘s study to assess the cellular uptake and internalization kinetics and intracellular distribution of diatomite-based porous biosilica NPs in a lung epidermoid carcinoma cancer cell line (H1355). The results obtained after 72 h showed high conformity with confocal fluorescence microscopy and photoluminescence measurements. Since this methodology does not harm the cell morphology or viability, diatomite-based NPs were distinguished even after 72 h. Raman bands correlated with diatomite-based NPs, and the cellular components confirmed the internalization and colocalization of nanocarriers with lipid vesicles. Fig. 10a and b exhibit conventional transmission optical images and the Raman image of cells incubated for 24 h with a nanocarrier. The possibility of reconstructing a false color Raman map of the H1355 cell is conceivable using the Multivariate Curve Resolution (MCR) method. Fig. 10c presents the Raman spectra, and the main spectral regions and related characteristics are concisely given in Table 5. The protein bands dominate the spectral bands of the nucleus, cytoplasm, and vesicle, accompanied by diatomite-based NPs, while the main component in the cellular areas is protein. The cell nucleus spectrum presented with a blue color demonstrates attenuated bands related to nucleic acids at 785, 1095, and 1575 cm^−1^. The most intense Raman peaks of the lipid are associated with the vibration of hydrocarbon chains. These spectral bands resemble intracellular vesicles like endosomes. The green-colored spectrum connected with vesicles accompanied by diatomite-based NPs shows that the significant spectral characteristics of diatomite-based NPs are evident below 600 cm^−1^ and superimposed with the spectral characteristics of lipids, predicting the encapsulation procedure of diatomite-based NPs by endosomes.^288^

**Table 5:** The positions of the characteristic Raman band regions in the cell spectra^a^

Band position (cm^−1^)	Assignment	Vibrational bonds	Indicative of
780–790	Pyrimidine bases	Ring breathing	Nucleic acid
1005	Phenylalanine	Symmetric stretching	Proteins
1095	O–P–O	Symmetric stretching	Nucleic acids backbone, phospholipids
1270–1350	Amide III	CH/NH deformation	Proteins
1425–1480	CH_2_/CH_3_	Deformation	Proteins/lipids
1650–1670	Amide I	C=O stretching	Proteins
1735	C=O	Stretching	Lipids
2800–3020	CH_3_, CH_2_, CH	Stretching	Lipids, proteins, other

a This table was adapted by permission from: *J. Biophotonics*, 2018, **11**, e201700207 (ref. 288)

Confocal fluorescence microscopy is a label-based technique to pursue the cellular uptake of diatomite-based NPs by labeling the diatomite-based NPs, cell membrane, and vesicles using Alexa Fluor® 488, WGA Alexa Fluor® 555, and Hoechst 33342, respectively. Furthermore, the cellular uptake kinetics and efficacy of the labeled siRNA-diatomite-based NPs were investigated for different incubation times, *i.e.*, 6, 18, 24, 48, and 72 h. Fig. 10d presents the confocal fluorescence microscopy, and the cellular uptake of label-free siRNA-diatomite-based NPs was appraised with Raman microscopic imaging under similar conditions. Confocal microscopy results for an accurate localization of diatomite-based NPs in H1355 cancer cells show that very few cells demonstrate the localization of diatomite-based NPs in the cell environment after 6 h with a slight cell uptake. The characteristic Raman spectra of lipid and diatomite-based NPs are not overlapped in the case with no internalization, as shown by the light blue dots in Fig. 10e at 6 h. However, the diatomite-based NPs cluster in. aggregations on the cell membrane, and no internalization can be observed. Increasing the incubation time from 6 h to 18 h increases the cellular uptake and diatomite-based NPs clusters. The diatomite-based NPs remain in the cell while an increased amount of accumulation of diatomite-based NPs around the cell nucleus is perceived.^288^

Additionally, Nakabayashi *et al.* studied advances in intracellular periphery and cell cycle by Raman imaging. Biomolecule aggregation inside the cells is known as molecular crowding in which alterations in the cell nucleus directly influence the stability and adjustments of the biomolecule. This technique quantitatively investigates the intracellular crowding periphery due to the intensity among C–H and O–H stretching bands.^289^

As reviewed in the last section, two general label-free and label-based methods have been considered due to the importance of detecting the cellular uptake and internalization of NPs. Label-free techniques (TEM, Raman microscopy, *etc.*) do not need a fluorophore. In contrast, label-based methods, like confocal fluorescent microscopy, are based on the fluorescent signal emitted from NPs due to their intrinsic property or a fluorophore–NP combination. The abbreviations used in the text and their related explanations are given in Table 6.

**Table 6:** The abbreviations used in the context and their definitions

Abbreviation	Definition
NPs	Nanoparticles
MNPs	Magnetic nanoparticles
GNPs	Gold nanoparticles
SNPs	Silver nanoparticles
UCNPs	Upconversion nanoparticles
MSNs	Mesoporous silica nanoparticles
IOH NPS	Inorganic–organic hybrid nanoparticles
BMP NPs	Betamethasone phosphate nanoparticles
CNCs	Cellulose nanocrystals
HNTs	Halloysite nanotubes
hNFs	Hybrid nanoflowers
MNCs	Magnetic nanoclusters
CNTs	Carbon nanotubes
CTAB	Cetyltrimethylammonium bromide
CTAC	Hexadecyltrimethylammonium chloride
NOPs	Nanooctopods
SWCNTs	Single-walled carbon nanotubes
QDs	Quantum dots
DLC	Drug loading content
DLE	Drug loading efficiency
CDSA	Crystallization-driven self-assembly
EPR	Enhanced permeability and retention
MH-S	A murine alveolar macrophage cell line
AR	Aspect ratio
OA	Oleic acid
CPPs	Cell-penetrating peptides
PLGA	Poly(lactic-*co*-glycolic acid)
PSMA	Poly(styrene-co-maleic anhydride)
PEG	Polyethylene glycol
PS	Polystyrene
PVP	Polyvinylpyrrolidone
PAMAM	Polyamidoamine
FMN	Flavin mononucleotide
PC	Phosphatidylcholine
HA	Hyaluronic acid
ATRA	All-trans retinoic acid
SA	Sialic acid
PBA	Phenylboronic acid
Tf	Transferrin
TQ	Thymoquinone
DOX	Doxorubicin
IBU	Ibuprofen
β-CD_3_	β-Cyclodextrin trimer
BN	Boron nitride
CS	Chitosan
AA	Anisamide
PTK-7	Protein tyrosine kinase-7
Fabs	Antigen-binding fragments
mAbs	Monoclonal antibodies
HSA	Human serum albumin
EGFR	Epidermal growth factor receptor
MDC	Mono-dansyl-cadaverine
BD	BODIPY fluorophore
MCR	Multivariate curve resolution
LSPR	Localized surface plasmon resonance
SERS	Surface enhanced Raman scattering
NIR	Near infra-red
DLS	Dynamic light scattering

### 6.5. Single-particle tracking

Single-particle tracking (SPT), a reliable method for directly differentiating the particular properties and dynamics of individual objects, has recently received a lot of attention with advances in optical microscopic methods.^290,291^ Typically, an image processing program analyzes the motion of the object after capturing it using an optical microscopic approach during an SPT measurement. Time-resolved trajectories of individual items are often chaotic and varied, but statistical characteristics like spatial displacement can provide a wealth of kinetic information. Numerous biological processes may be explained in detail, such as the interaction between a virus and its host and also the intracellular internalization mechanism of a drug cargo.^292^ Fluorescence-based and nonfluorescence-based (*e.g.*, scattering-based) SPT technologies can be broadly divided into two groups depending on the types of probe used for SPT, such as fluorescent molecules and plasmonic nanostructures.^293,296^ Due to their excellent sensitivity and specificity, fluorescent probes have been widely utilized in various fields. Among other drawbacks, poor photostability and low quantum yield may prevent their use in long-term monitoring. Plasmonic nanostructures, unlike fluorescent probes, have distinctive physicochemical characteristics, such as a large extinction cross-section, good biocompatibility, and optimal photostability, which make them the best candidates for studying diffusion behavior on the cell membrane and inside the cell for an extended period.^297–301^ Additionally, in recent years, various scattering-based optical microscopic methods have been developed that use plasmonic nanostructures as the probe.^302–304^

#### 6.5.1. Plasmonic probe for SPT

The right probe is one of the most critical components of an SPT, and its physicochemical and optical characteristics significantly influence the accuracy of SPT results. Due to its distinctive LSPR effect, plasmonic nanostructures of various sizes and morphologies are the most frequently employed probes based on light scattering.^305–307^ The LSPR is adjustable and dependent on the size, shape, material, and environment around the plasmonic nanostructure, as previously mentioned. One might alter one of these variables to meet experimental needs. Among these plasmonic nanostructures, noble metal nanostructures, such as Au and Ag NPs, have been widely used in SPT.^294,295^

#### 6.5.2. Optical microscopic imaging methods for scattering-based SPT

##### 6.5.2.1. Dark-field optical microscopy (DFM)

DFM is one of the most widely used optical methods for tracking and detecting plasmonic NPs in biological samples. Collecting scattered light from the plasmonic NPs is essential for the production of DFM images.^290^ To meet various needs, DFM is typically paired with other pieces of equipment. For instance, the LSPR spectrum shifts from the plasmonic NPs may be seen at the single-particle level by combining DFM with a spectrometer.^308^ According to DFM and scattering spectroscopy, a nanosensor (Au@Cu core–shell NPs) as a real-time optical probe to detect decreased nicotinamide adenine dinucleotide (NADH) was proposed.^309^ The intensity ratio in the two detection channels may be used to compute the two-dimensional (2D) orientation of a single anisotropic nanoparticle. For scattering-based SPT, DFM is a popular and effective approach for examining the optical characteristics of plasmonic NPs.

##### 6.5.2.2. Total internal reflection scattering microscopy (TIRSM)

TIRSM, derived from total internal reflection fluorescence (TIRF) microscopy, has recently been created to significantly enhance the sensitivity and signal-to-noise ratio (SNR) of SPTs. Prisms or objective lenses are commonly used to achieve total internal reflection scattering (TIRS) lighting.^290^ In a prism-type TIRSM, the sample is excited by an evanescent wave. Only things placed a few hundred nanometers away from the cover glass are excited by the evanescent wave. By utilizing this technique, Ye *et al.* could accurately calculate the three-dimensional (3D) angle data on a single gold nanorod (GNR) near the liquid/solid border.^290,310^ Only the probe at the interface could be excited for TIR illumination because the excitation wavelength was chosen to match the longitudinal resonance of the GNR, significantly improving the selectivity and sensitivity toward the plasmonic probe. It is simple to estimate 3D angle information and accurate spatial localization by altering the polarization of the incident light.

##### 6.5.2.3. Interferometric scattering (iSCAT) microscopy

Another scattering-based imaging technique that is effective for high-speed SPT is iSCAT microscopy.^311–313^ iSCAT microscopy has outstanding sensitivity and resolution for monitoring tiny NPs.^314^ The minimum size for traditional scattering-based imaging technologies (*e.g.*, DFM) is roughly 30 nm, since the scattering signal of small particles rapidly decreases as the sixth power of the particle diameter.^315^ Small particle identification is achievable since the generated signals are proportional to the scattered field amplitude and only get weaker by three times the particle diameter.^313^ The SNR of iSCAT is proportional to N_1,2_ for shot-noise-limited experiments, where N is the observed number of photons per unit time. A high SNR may be attained with a brief integration time by modulating the laser power.^316^

##### 6.5.2.4. Differential interference contrast microscopy (DICM)

Another well-liked approach for imaging plasmonic NPs and cell organelles is DICM with unique imaging contrast. The contrast of the differential interference (DIC) image depends on the gradient of the refractive index in various specimen regions.^317,318^ The intensity of a DIC image reveals bright and dark details due to the various refractive indices and thicknesses of the material, which enhance the contrast in DICM images. This technique allows for the decoding of the complete 3D orientation data for a single GNR in the four quadrants of the Cartesian plane.^319^ High spatial and temporal resolution is used to position the exact azimuthal and polar angles of the GNRs in the focal plane. Additionally, the rotational dynamics of a nanocargo on the cell membrane have been effectively studied using DICM.^320^

## 7. Future outlook

This review has demonstrated that patterns, albeit sometimes uncertain, are continuously arising. However, the effect of AR on NP uptake remains unsolved. The difference could be the result of difficulties in producing NPs with one physical–chemical parameter changing at a time; for instance, alterations in surface charge may affect the hydrophobicity. Accordingly, to clarify the ascribed property, either more exact experiments with one parameter varied at a time are required, or a multivariate route could be applied. In the latter instance, all NP characteristics that may be altered have to be measured and reported in the future to provide a complete view of causes and effects. Generally, many contradictory data and results from various reports were perused in this study. The restricted number of NP features and the different experimental conditions examined resulted in uncertainties in the interpretation of some results. Information about NP properties that characterize accumulation is limited to data from toxicological reports.

Specifically, the effect of NP shape on cellular uptake is still unresolved. This is due to a lack of commercially available approaches to produce nonspherical NPs and limited procedures to obtain NPs with only one property changed at a time. The exact reciprocation of shape and the attributed determinant (length, volume, or surface area) for NPs with constant surface chemistry need experimental assessment. Many studies have presumed that particles are not aggregated. Particle size is often evaluated in pure NP solution and their shelf life is detected. Future enhancements will benefit from reports accomplished with standardized protocols for cell types and experimental setups, to defeat incomparability between reports carried out in various laboratories and to manifest more data about the underlying mechanism of particle uptake by cells. Standardized experimental approaches would possibly fill knowledge gaps and resolve apparent contradictions. Finally, extrapolation between different NPs and between different experimental conditions is required. In this case, modeling is inevitable.

## 8. Conclusion

In the last few decades, due to the extensive use of NPs in biomedical fields, methods for functionalizing NPs have evolved. Developments in the modification of the size, shape, and surface of NPs included specific cancerous cell targets. Furthermore, the adjustability of the physicochemical features of NPs according to the characteristics of cancer cells has expanded the use of these nanocarriers not only for drug delivery and cancer treatment but also for diagnosing these cells. Cellular uptake, internalization, and cytotoxicity are the most significant issues related to an optimized nanocarrier in the biomedical area. Hence, surface modification and optimization of a nanocarrier is a potent tool for upgrading cell internalization and biocompatibility that has been authenticated in many publications.

Additionally, while using particular molecules as surface modifiers, the active and passive uptake processes would be improved and *in vivo* cytotoxicity diminished, leading to suitable treatment. In addition to surface modification, various shape and size controlling parameters, including pH, solvents and synthesis routes, have been discussed in depth. Diverse cellular uptake and internalization mechanisms were debated. The effect of NP morphology and size on the internalization procedure was more critical in recent research. Different techniques related to the detection of nanocarriers in the cellular uptake process were investigated. Overall, according to the concepts reviewed, the rod-shaped morphology with a narrow cross-section is considered the most likely to penetrate the cell membrane among all shape classifications of drug nanocarriers. Moreover, several studies have focused on various factors to produce nanorods with optimized size and AR. Additionally, the orientation of non-spherical nanocarriers, such as nanorods, could affect the cellular uptake process, while nanocarriers with a tangent angle of less than 45° better penetrate the membrane, and Ω = 90° is beneficial. These nanocarriers show different behaviors when confronting the cells of diverse organs whose fields should be investigated in future studies.

## Conflicts of interest

The authors declare no conflict of interest.

## Supplementary Material
