# Correction: Effects of morphology and size of nanoscale drug carriers on cellular uptake and internalization process: a review

**DOI:** 10.1039/d5ra90027a

**Published:** 2025-03-13

**Authors:** Wenjie Zhang, Reza Taheri-Ledari, Fatemeh Ganjali, Seyedeh Shadi Mirmohammadi, Fateme Sadat Qazi, Mahdi Saeidirad, Amir KashtiAray, Simindokht Zarei-Shokat, Ye Tian, Ali Maleki

**Affiliations:** a Department of Nuclear Medicine, West China Hospital, Sichuan University No. 37, Guoxue Alley Chengdu 610041 Sichuan Province P. R. China; b Catalysts and Organic Synthesis Research Laboratory, Department of Chemistry, Iran University of Science and Technology Tehran 16846-13114 Iran Rezataheri13661206@gmail.com R_taheri94@alumni.iust.ac.ir maleki@iust.ac.ir +98 21 73021584 +98 21 77240640-50; c Department of Orthodontics, West China Hospital of Stomatology, Sichuan University No. 14, 3rd Section of South Renmin Road Chengdu 610041 P. R. China

## Abstract

Correction for ‘Effects of morphology and size of nanoscale drug carriers on cellular uptake and internalization process: a review’ by Wenjie Zhang *et al.*, *RSC Adv.*, 2023, **13**, 80–114, https://doi.org/10.1039/D2RA06888E.

This correction is being published to alert readers that Fig. 7(i)–(t) was reproduced from an article in *Small*, which has now been retracted.^1^ The article was cited as ref. 8 of this *RSC Advances* article.

The retraction notice of the *Small* article states that “investigation uncovered inappropriate duplication of images (panels of Figures 7d and 12a) between this and other articles that were previously published in a different scientific context elsewhere.”

Fig. 7(d) of the *Small* article was reproduced as Fig. 7(i)–(t) of this *RSC Advances* article.

The Royal Society of Chemistry apologises for these errors and any consequent inconvenience to authors and readers.
